# GPR35 prevents osmotic stress induced cell damage

**DOI:** 10.1038/s42003-025-07848-9

**Published:** 2025-03-22

**Authors:** Joshua E. Elias, Mekdes Debela, Gavin W. Sewell, Richard J. Stopforth, Hannah Partl, Sophie Heissbauer, Lorraine M. Holland, Tom H. Karlsen, Arthur Kaser, Nicole C. Kaneider

**Affiliations:** 1 Cambridge Institute of Therapeutic Immunology and Infectious Disease, University of Cambridge, Cambridge, CB2 0AW UK; 2 Division of Gastroenterology and Hepatology, Department of Medicine, University of Cambridge, Cambridge, CB2 0QQ UK; 3 Division of Surgery, Inflammatory Diseases and Transplantation, Oslo University Hospital, Oslo, Norway

**Keywords:** Mechanisms of disease, Gastrointestinal cancer, Cell growth

## Abstract

GPR35 is an orphan G-protein coupled receptor that has been implicated in the development of cancer. GPR35 regulates the Na^+^/K^+^-ATPase’s pump and signalling function. Here we show GPR35’s critical role in ion flux that in turn controls cellular osmotic pressure and Na^+^-dependent transport in HepG2 and SW480 cells. GPR35 deficiency results in increased levels of intracellular Na^+^, osmotic stress and changes in osmolytes leading to increased cells size and decreased glutamine import in vitro and in vivo. The GPR35-T108M risk variant, which increases risk for primary sclerosing cholangitis and inflammatory bowel disease, leads to lower intracellular Na^+^ levels, and enhanced glutamine uptake. High salt diet (HSD) in wildtype mice resembles the intestinal epithelial phenotype of their *Gpr35*^−/−^ littermates with decreased Goblet cell size and numbers. This indicates that GPR35’s regulation of the Na^+^/K^+^-ATPase controls ion homeostasis, osmosis and Na^+^-dependent transporters.

## Abstract (web summary)

GPR35 controls the transmembrane ion gradient by regulating Na/K-ATPase function with knock-on effects on osmosis, cellular stress and glutamine uptake.

## Introduction

Salt is an essential component of the human diet and its uptake vital to maintain physiological electrolyte levels. Salt has been a precious ingredient to season and preserve food for thousands of years and has, in fact, shaped human history. Easy access to dietary salt in the present time, and the abundance of salt in processed foods, has led to its association with multiple diseases linked to ‘Westernisation’^1^.

Absorption of water, nutrients and electrolytes (mainly Na^+^, Cl^−^ and K^+^) occurs throughout the small intestine, and ~1500 mL of remaining liquid is absorbed in the colon per day^2^. Na^+^ coupled co-transport plays a vital role in nutrient and electrolyte uptake from the gut lumen. This is mainly achieved by the active basolateral efflux of Na^+^ facilitated by the Na^+^/K^+^-ATPase^3^ whereby K^+^ accumulates intracellularly, and then exits the cell actively via the H^+^/K^+^-ATPase and basolateral K^+^ channels^4^. Importantly, by pumping K^+^ across the cell membrane, the Na^+^/K^+^-ATPase is quintessential in maintaining the membrane potential in intestinal epithelial cells^5^. This continuous in- and efflux of ions poses a challenge to the cell’s volume control mechanisms, which is tightly regulated to allow cell survival and proliferation^6^. Although the downstream pathways and ion channels involved in volume control have been well characterised^7^, the upstream sensing and signalling mechanism has remained elusive.

Hyperosmolarity is associated with a variety of intestinal inflammatory conditions, such as Crohn’s disease^8^ and ulcerative colitis (UC)^9^. The incidence of Crohn’s disease has increased >10-fold over the last 50 years in the Western world. Its prevalence is increasing worldwide, including in populations previously considered at low risk. This rise obviously cannot be explained by genetics, and environmental factors, such as a diet high in sodium therefore need to be considered^10^. High salt was shown to induce the production of proinflammatory cytokines and hence contribute to inflammatory reactions in the gut^11,12^. A high salt diet has also been mechanistically linked to increased Th17 immune responses in the intestine that drive mucosal inflammation^13,14^.

G-protein coupled receptor 35 (GPR35) is an orphan G-protein coupled receptor (GPCR) highly expressed on the intestinal epithelium and mononuclear phagocytes^15,16^. We have discovered that GPR35 interacts with the Na^+^/K^+^-ATPase and promotes its ion transport function in ligand-independent manner, i.e. unaffected by pharmacological agonists and antagonists, hence demonstrating a non-canonical function of this receptor^17^. Lack of GPR35 led to a decrease in uptake of Rubidium (Rb^+^), a proxy of K^+^, in macrophages and Caco-2 cells. GPR35’s polymorphic variant T108M, which is associated with increased risk for ulcerative colitis^18,19^, Crohn’s disease, and primary sclerosing cholangitis^20^, increased Na^+^/K^+^-ATPase’s pump activity^17^. In addition to its pump function, Na^+^/K^+^-ATPase also serves as scaffold and activator for Src kinase^21^. GPR35 promoted intestinal epithelial cell proliferation by phosphorylating Src kinase in a Na^+^/K^+^-ATPase-specific manner. GPR35 increased intestinal tumour formation by promoting tumour cell proliferation and tumour angiogenesis^17,22^. GPR35 mRNA and protein expression is indeed upregulated in a number of cancers, including colorectal, liver and breast^23,24^. Higher GPR35 expression has been associated with poorer prognosis in colorectal cancer^23^.

The endogenous, physiological ligand of GPR35 has still not been identified despite substantial efforts. One proposed candidate is kynurenic acid, albeit a several-log difference in *K*_d_ between human and rodent GPR35 naturally casts doubts^25^. Regardless, there is intriguing evidence that kynurenic acid at the (notably high and pharmacological) concentration of 10 mM triggers the translocation of GPR35 to the outer mitochondrial membrane^26^. GPR35 then interacts with ATPIF1 on the mitochondrial outer membrane of cardiac myocytes and thereby induces ATP synthase dimerization, which prevents ATP loss under ischaemic conditions^26^. This work also suggested that there may be an interaction between GPR35 and the voltage-dependent anion channel (VDAC) on mitochondrial membranes^26^.

Here we show that GPR35 regulates intracellular ion concentrations by modulating Na^+^/K^+^-ATPase activity, with consequences for cell size, macro-nutrient uptake and cellular proliferation. Loss of GPR35 leads to increased intracellular Na^+^ concentrations and this imbalance causes osmotic stress as demonstrated by p38 MAPK activation, and a compensatory decrease in endogenous osmolytes. The PSC-IBD risk-variant GPR35-T108M displays lower intracellular potassium levels and increased uptake of glutamine, consistent with hyper-morphic function. In addition to decreased Src-kinase activation previously reported^17^, diminished Na^+^-gradient-dependent glutamine uptake contributes to impaired cellular proliferation in *GPR35* knock-down cells. Notably, levels of endogenous osmolytes in biopsies of patients with active UC and PSC were substantially lower compared to healthy controls, suggesting a pathophysiological role of this mechanism. These results suggest that epithelial ion homoeostasis and osmotic stress may be an unrecognised primary pathogenic mechanism in inflammatory bowel disease.

## Results

### GPR35 regulates osmosis

**Fig. 1:**
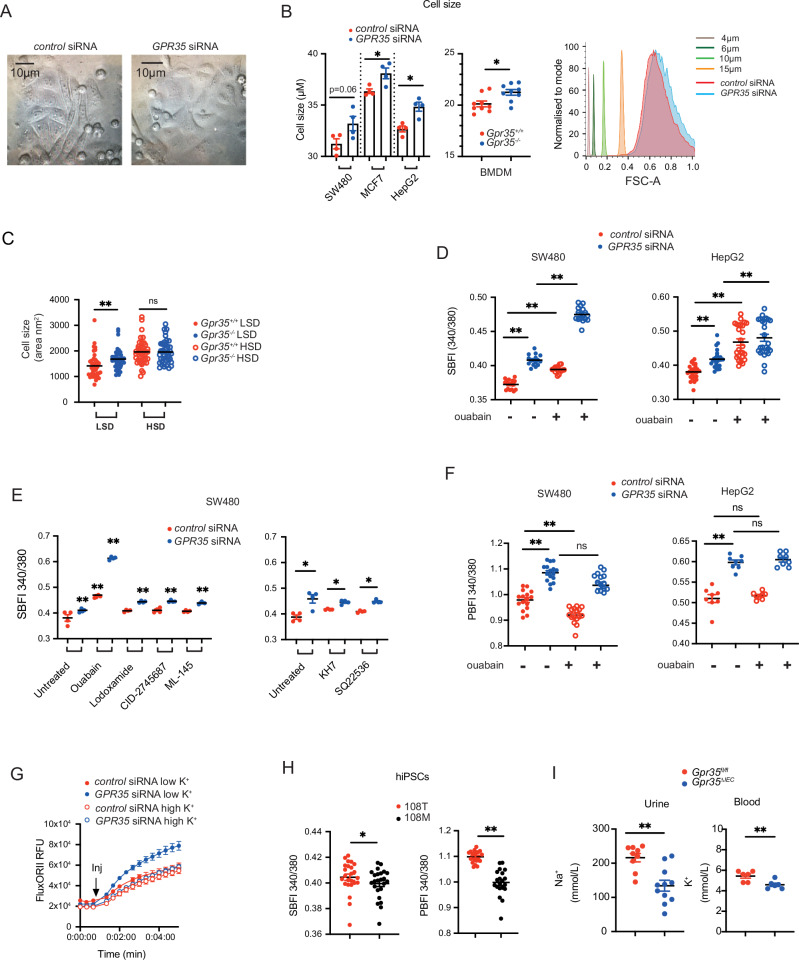
Lack of GPR35 results in changed cell size and ion homoeostasis. **A** Knock down of GPR35 leads to ballooning and reduced growth of HepG2 cells. **B** Knock-down of GPR35 in human cancer cell lines leads to an increase of size. Bone marrow derived macrophages from *Gpr35*^−/−^ mice were increased compared to *Gpr35*^*+/+*^ cells. Size was measured using flow cytometry. Size was calculated using forward scatter and size reference beads. *N* = 4 for cell lines. **C** Mice were on a low salt diet (LSD) or high salt diet (HSD). Cell diameters were measured and area was calculated from H&E stained intestinal samples. *N* = 42 (from 6 mice per group) **D** Intracellular Na^+^ levels measured by fluorescence (SBFI), ouabain used as control inhibits the Na^+^/K^+^-ATPase. Left panel SW480 intestinal epithelial cells, right panel HepG2 hepatic epithelial cells. *N* = 16. **E** Left panel. Measurement of intracellular Na^+^ (SFBI) with canonical GPR35 agonists or antagonists, ouabain as control. Right panel. Measurement of intracellular Na^+^ (SFBI) with adenyl cyclase inhibitors KH7 or SQ22536. *N* = 4. **F** Intracellular K^+^ levels measured by fluorescence (PBFI), ouabain used as control inhibits the Na^+^/K^+^-ATPase. Left panel SW480 intestinal epithelial cells, right panel HepG2 hepatic epithelial cells. *N* = 16. **G** Measurement of K^+^-flux by Tl assay over 5 min. *N* = 4. **H** Intracellular Na^+^ and K^+^ levels in hiPSC carrying the risk or non-risk variant of GPR35. Measured by fluorescence (SBFI for Na^+^ and PBFI for K^+^). *N* = 17 to 25. **I** Urine and blood levels of Na^+^ and K^+^ in *Gpr35*^*fl/fl*^ *and Gpr35*^*DIEC*^ mice. N = 9 –11. Data represented as mean ± s.e.m. Statistical significance was calculated using Mann Whitney *U* after Kruskal Wallis testing. *p < 0.05, **p < 0.01.

To assess GPR35’s impact on ion homoeostasis, we used siRNA to knock down the receptor in a range of GPR35-expressing cancer cell lines. To our surprise, this profoundly affected the cells’ morphology as cells appeared larger and swollen (Fig. 1A). FACS analyses confirmed that the size of cell lines SW480, MCF7 and HepG2 increased in *GPR35*- compared to *control*-silenced cells (Fig. 1B). We observed a similar increase in size in bone marrow-derived macrophages from *Gpr35*^–/–^ compared to *Gpr35*^*+/+*^ mice (Fig. 1B). The area of intestinal epithelial cells in *Gpr35*^–/–^ mice compared to their *Gpr35*^+/+^ littermates was increased (Fig. 1C). Intriguingly, feeding mice a high salt diet (HSD) led to larger epithelial cells in *Gpr35*^+/+^ mice, reaching similar sizes as observed in HSD-fed *Gpr35*^–/–^ mice (Fig. 1C).

We hypothesised that the increase in cell size in GPR35-deficient cells might be related to decreased Na^+^/K^+^-ATPase activity resulting in changes in intracellular Na^+^ levels. We therefore measured cellular Na^+^ concentration via the specific, ratiometric fluorescent probe SBFI. Indeed, *GPR35*-silenced SW480 and HepG2 cells exhibited markedly elevated fluorescence, indicative of increased intracellular Na^+^ compared to control-silenced cells (Fig. 1D). Inhibition of the Na^+^/K^+^-ATPase with ouabain increased intracellular Na^+^ even further in both SW480 and HepG2 cells (Fig. 1D). We repeated these experiments in the presence of the GPR35 agonist lodoxamide, and the antagonists CID2745687 and ML-145, to see whether canonical GPR35 signalling played a role in ion flux. None of these pharmacological ligands changed the intracellular Na^+^ levels in *control*- or *GPR35* silenced SW480 cells. Neither did the adenylyl cyclase inhibitors SQ22536 or KH7 that are involved in G protein-coupled signalling impact on Na^+^ levels (Fig. 1D,E). The Na^+^/K^+^ inhibitor ouabain, used as a positive control, increased intracellular Na^+^ levels in both *control*- and *GPR35*-silenced cells (Fig. 1D,E). Blocking the Na^+^/K^+^-ATPase in cells silenced for *GPR35* has an even larger effect on intracellular Na^+^ levels. Although *GPR35* siRNA treatment led to a 75% knockdown in mRNA^17^ ouabain treatment may have an additive effect.

These results indicated that the interaction between GPR35 and the Na^+^/K^+^ATPase may not involve canonical GPCR signalling. It pointed to direct modulation of intracellular Na^+^ by Na^+^/K^+^-ATPase in a GPR35 ligand-independent manner. Interestingly, cellular K^+^ levels were also elevated in *GPR35*- compared to *control*-silenced HepG2 and SW480 cells, as measured by the K^+^-selective ratiometric probe PBFI (Fig. 1F). PBFI is 1.5-fold more selective for K^+^ than for Na^+^, and usually this selectivity is sufficient to discriminate K^+^ from Na^+^ because intracellular K^+^ concentrations are ~10 times higher than Na^+^ concentrations^27^. We had previously demonstrated reduced Na^+^/K^+^-ATPase-dependent uptake of Rb^+^ in *Gpr35*^–/–^ cells^17^. Rb^+^ serves as proxy for measuring K^+^ uptake by Na^+^/K^+^-ATPase due to its very similar biophysical properties and its absence from biological systems. Rb^+^ uptake, however, does not assess release from intracellular K^+^ stores. There are also slight differences how Rb^+^ and K^+^ is transported through K^+^ channels^28^. Elevated K^+^ levels in *GPR35* silenced SW480 and HepG2 cells hence may be a knock-on indirect effect, potentially compensating for decreased uptake from extracellular space in the absence of GPR35. To maintain K^+^ homoeostasis within the cell, K^+^ exits cells via outward rectifying channels^29^ and enters cells by high conductance K^+^ channels which are activated by high Na^+^ and low K^+^^30^. The activity of high conductance K^+^ channels can be measured via the FluxOR dye. This dye generates a fluorescent signal upon binding of thallium ions (Tl^+^), which act as another surrogate for K^+^, entering through open K^+^ channels^31^. Upon a Tl^+^ pulse, FluxOR fluorescence markedly increased in *GPR35*-silenced compared to control-silenced SW480 cells (Fig. 1G). This was completely reversed by pre-incubation with a buffer high in K^+^ (125 mM), which partially depolarises the cell membrane and thereby inactivates these channels (Fig. 1G). This suggests that cells lacking GPR35 compensate for decreased Na^+^/K^+^-ATPase activity by increasing the inwards flux of K^+^ via these channels.

GPR35’s role in K^+^ regulation was corroborated by ion levels and -fluxes in KOLF-2 human induced pluripotent stem cells (hiPSCs) gene-edited to express either the PSC/UC-associated GPR35-108M, or the non-risk −108T variant. Intracellular Na^+^ levels were lower in 108 M mutants (Fig. 1H), as were K^+^ levels (Fig. 1H). These observations were a mirror image of results from GPR35-deficient cells and hence corroborated that the disease risk-associated −108 M variant was hypermorphic. We concluded that these direct and compensatory alterations in intracellular ion homoeostasis might cause the gross phenotypic size changes in *GPR35*-silenced SW480 cells (Fig. 1A).

To investigate whether GPR35-dependent perturbation of intracellular ion homoeostasis has systemic consequences, we measured Na^+^ and K^+^ levels in blood and urine of mice with selective *Gpr35* deletion in their intestinal epithelium (*Gpr35*^fl/fl^;*Vil*-Cre^+^, ‘*Gpr35*^ΔIEC^’ mice). Na^+^ levels were lower in the urine of *Gpr35*^ΔIEC^ mice compared to their *Gpr35*^fl/fl^ littermates (Fig. 1I), but *Gpr35*^ΔIEC^ mice exhibited lower blood K^+^ levels compared to *Gpr35*^fl/fl^ littermates (Fig. 1I). Importantly, GPR35 is not expressed in kidneys (https://www.proteinatlas.org/ENSG00000178623-GPR35/tissue), and the Villin promoter transgene used here does not leak in the tubular epithelium. Hence the changes in blood and urine levels of Na^+^ and K^+^ in *Gpr35*^ΔIEC^ mice indicate systemic consequences of altered ion transport by the intestinal epithelium.

### GPR35 deficiency causes osmotic stress

**Fig. 2:**
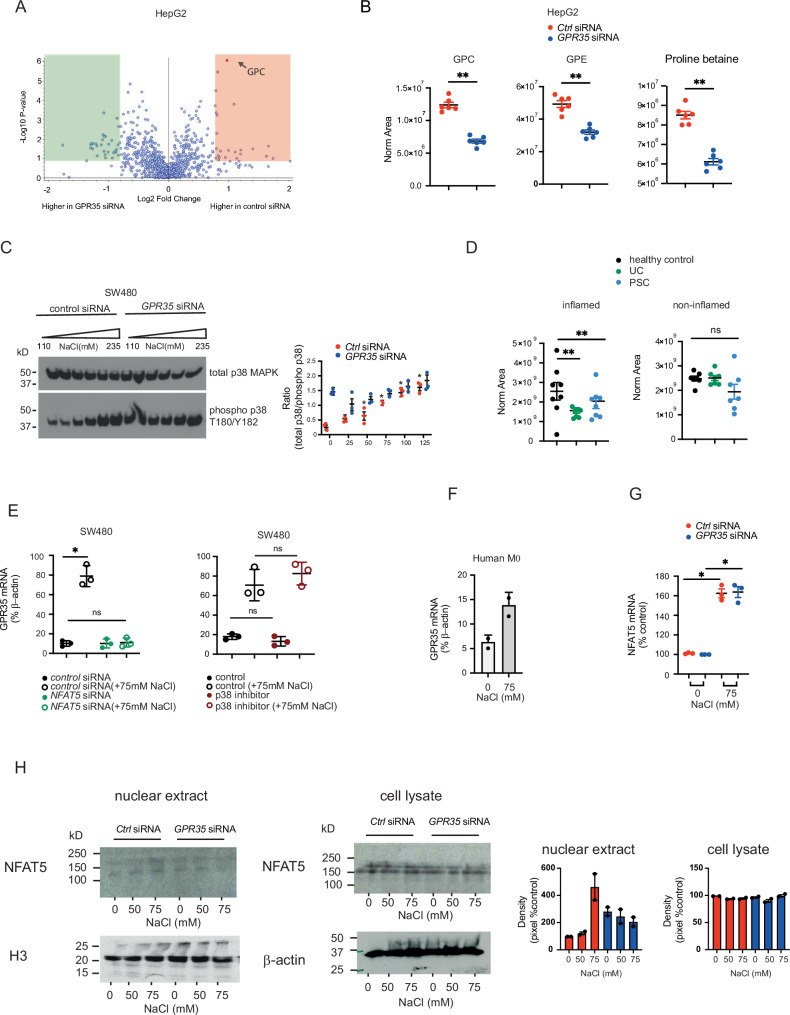
GPR35 regulates intracellular osmotic pressure. **A** Metabolomic screen of *GPR35* silenced and control HepG2 cells. Data are shown as log fold change. Measured by mass spectrometry. *N* = 3. **B** Osmolytes were measured by mass spectrometry in control and *GPR35* silenced HepG2 cells. *N* = 6. **C** Western blot analysis of p38MAPK activation with addition of 25 to 125 mM of NaCl. Western blot band density was calculated using ImageJ. *N* = 3 **D** The endogenous osmolyte GPC was measured by mass spectrometry in colonic biopsies of healthy control, UC or PSC patients. *N* = 7-8 **E** Right panel: *GPR35* mRNA levels under normo- or hypersaline conditions with and without *NFAT5* siRNA. Left panel: *GPR35* mRNA levels under normo- or hypersaline conditions with and without p38 MAPK inhibitor. *N* = 3. **F***GPR35* mRNA levels in human macrophages under normo- or hypersaline conditions. *N* = 2. **G***NFAT5* mRNA levels under normo- or hypersaline conditions with and without *GPR35* siRNA. *N* = 3. **H** Western blot analysis of NFAT5 in cell lysates and nuclear extracts. *N* = 5. Western blot band density was calculated using ImageJ. All data represented as mean ± s.e.m. Statistical significance was calculated using Mann Whitney *U* after Kruskal Wallis testing. *p < 0.05, **p < 0.01.

Loss of GPR35’s regulatory function on Na^+^/K^+^-ATPase activity with consequent perturbation in intracellular ion levels may lead to osmotic stress. Cells balance osmotic pressure against the extracellular environment by adapting intracellular ion concentrations, and by accumulating or exporting compatible organic osmolytes such as glycerophosphocholine (GPC)^32^. GPC is synthesised from phosphatidylcholine when extracellular levels of Na^+^ are high^33^. GPC as an osmolyte is particularly well studied in kidney cells, but it can accumulate to high levels in most cells without perturbing intracellular signalling, protein folding or DNA integrity^34^. An unbiased high-complexity metabolomic comparison of aqueous extracts of *GPR35*- with control-silenced HepG2 cells indeed identified GPC as its top hit (Fig. 2A). Levels of GPC, and of the fellow organic osmolytes glycerophosphoethanolamine (GPE) and proline betaine, were markedly lower in *GPR35*- compared to control-silenced HepG2 cells (Fig. 2B). This reduction in organic osmolytes would compensate for high osmotic pressure inside GPR35-deficient cells, and hence was consistent with GPR35 deficiency causing osmotic stress.

The stress-activated protein kinase p38 MAPK is phosphorylated in response to heat shock, UV radiation and osmotic stress^35–39^. Under baseline culture condition, T180/Y182 phosphorylation of p38 was increased in *GPR35*- compared to *control*-silenced SW480 cells (Fig. 2C and Supp Fig. 1). Increasing extracellular osmolality by adding NaCl to culture media dose-dependently increased p38 phosphorylation in *control*-silenced cells, reaching high levels observed in *Gpr35*-silenced cells at baseline (Fig. 2C and Supp Fig. 1). In contrast, increasing extracellular osmolality had no effect on p38 phosphorylation in *Gpr35*-silenced SW480 cells (Fig. 2C and Supp Fig. 1). This provided further evidence for osmotic stress in GPR35-deficient cells. The T108M polymorphism in GPR35 confers risk for inflammatory bowel disease and PSC. We collected colonic mucosal biopsies from patients with ulcerative colitis, PSC and healthy individuals undergoing colonoscopies. We quantified organic osmolytes by LC-MS, and observed lower levels of GPC in inflamed mucosa from patients with ulcerative colitis and PSC-IBD, compared to uninflamed samples from healthy controls (Fig. 2D). No difference in GPC levels were observed in non-inflamed mucosa (Fig. 2D). This suggests that the inflamed mucosa in ulcerative colitis exhibits signs of osmotic stress, offloading GPC in order to reduce intracellular osmotic pressure. Increased p38 MAPK phosphorylation in inflamed UC mucosa had previously been reported^40^. *Further work is required to ascertain whether this is a primary event and, if so, what agent is causing osmotic stress. There is some evidence however that the human intestinal epithelium is very susceptible to osmotic stress, especially when there is pre-existing inflammation present*^41^. This could fit with the prevailing hypothesis that IBD is triggered by an environmental stressor in genetically susceptible individuals.

### GPR35 expression is induced by osmotic stress via NFAT5

*GPR35* mRNA expression itself responded to extracellular hyper-osmolality. *GPR35* mRNA expression increased 5-fold in SW480 cells when 75 mM NaCl was added to isotonic (150 mM) culture medium for 12 h (Fig. 2E). Nuclear factor of activated T cells 5 (NFAT5) is a transcriptional regulator of the cellular response to osmotic stress^42^, and highly expressed in the gastrointestinal tract (https://www.proteinatlas.org/ENSG00000102908-NFAT5/tissue). NFAT5 is activated by osmotic stress, and its protein expression is increased upon NaCl exposure of intestinal cells^43^. Increased *GPR35* mRNA expression in SW480 cells under hyper-osmotic stress was completely abrogated upon silencing *NFAT5* expression, whilst not affecting baseline *GPR35* mRNA expression under isotonic conditions (Fig. 2E). Blocking p38 MAPK phosphorylation had no significant effect on *GPR35* mRNA expression (Fig. 2E). Human macrophages also upregulated *GPR35* mRNA expression when culture medium was rendered hyper-osmotic by adding 75 mM of NaCl (Fig. 2F). As expected, *NFAT5* mRNA expression increased in SW480 cells under hyper-osmotic conditions, which was not affected by *GPR35* silencing (Fig. 2G). Upon inducing hyper-osmolality by adding increasing amounts of NaCl, nuclear NFAT5 protein levels increased in control-silenced SW480 cells, but were already elevated at isotonic baseline in *GPR35*-silenced cells (Fig. 2H and Supp Fig. 1). Hence the NFAT5-mediated response to osmotic stress includes the induction of GPR35, whose absence itself causes osmotic stress and NFAT5 activation.

### GPR35 promotes Na^+^-dependent amino acid uptake

**Fig. 3:**
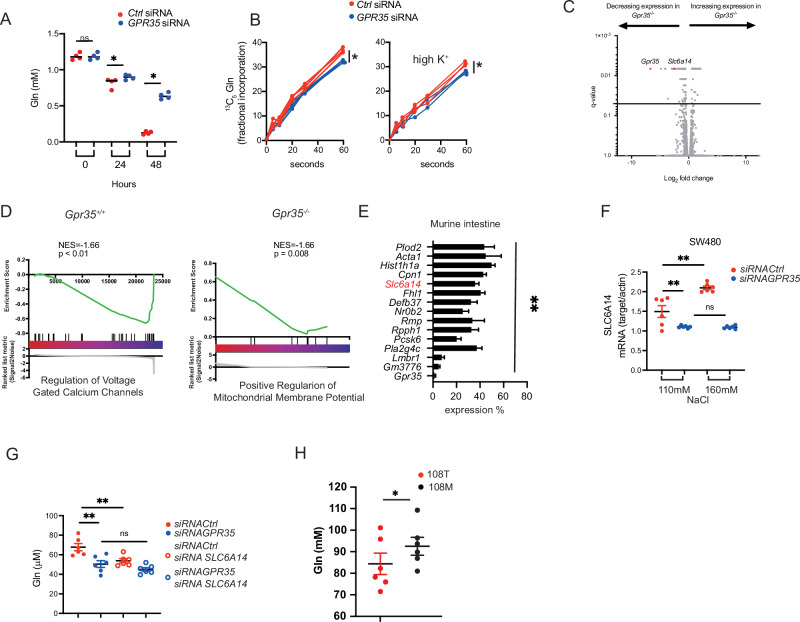
Na^+^-dependent glutamine uptake is regulated by GPR35. A. **A** Glutamine concentration in culture medium of SW480 cells at different time points of cell culture showing increased consumption of glutamine by control siRNA compared to GPR35 siRNA transfected cells. Measured by Promega glutamine GLO assay. *N* = 3. **B** [13C5] glutamine uptake over 60 s, measured by LC-MS, in SW480 cells 48 h following siRNA transfection. Left panel: Uptake under physiological K^+^ and Na^+^ conditions. right panel: Uptake under high K^+^ conditions (50 mM KCl). *N* = 3 **C** RNAseq of *Gpr35*^*+/+*^ and *Gpr35*^−/−^ murine ileum. N = 6 per genotype. **D** Pathway analysis of RNAseq in murine ileum of *Gpr35*^*+/+*^ and *Gpr35*^−/−^ mice. **E** RNAseq of *Gpr35*^*+/+*^ and *Gpr35*^−/−^ murine ileum. Data are shown as % gene expression wildtype mice. *N* = 5 mice per genotype. **F***SLC6A14* mRNA expression in SW480 cells under physiological and high Na^+^ conditions. *N* = 6. **G** Glutamine uptake in control or *GPR35* silenced SW480 cells with additional *SLC6A14* knock down. *N* = 6. **H** Glutamine uptake in hiPSC carrying the 108 T norm or the 108 M risk variant of GPR35. Intracellular glutamine was measured by luminescence. *N* = 6. All data represented as mean ± s.e.m. Statistical significance was calculated using Mann Whitney *U* after Kruskal Wallis testing. *p < 0.05, **p < 0.01.

Absorption of nutrients in the intestine requires an electrogenic gradient created by Na^+^/K^+^ATPase^7^. We hypothesised that GPR35 may facilitate nutrient uptake in specialised cell types such as the intestinal epithelium. Glutamine is the most abundant amino acid in the human body^44^, its transport across cell membranes is Na^+^-dependent^45^, and the intestinal epithelium is a net consumer of glutamine. We measured glutamine in supernatants from either *GPR35* or *control* silenced SW480 intestinal epithelial cells cultured over 48 h, and found markedly higher glutamine consumption in GPR35-expressing compared to GPR35-silenced cells (Fig. 3A). We therefore measured the actual uptake of stable isotope-labelled [^13^C_5_] glutamine over a 60 s period. [^13^C_5_] was indeed markedly increased in *control*- compared to *GPR35*-silenced SW480 cells, as evident by total and fractional incorporation of the [^13^C_5_] isotopomer (Fig. 3B). To test whether the [^13^C_5_] uptake was affected by K^+^ we repeated the experiment under 50 mM KCl conditions. GPR35 compared to control-silencing resulted in altered glutamine uptake in both conditions, ie under normal and high K^+^ conditions. Glutamine uptake was slightly decreased with 50 mM of KCl in the buffer (Fig. 3B).

Next we performed RNAseq analysis in intestinal biopsies of *Gpr35*^*+/+*^ and *Gpr35*^−/−^ mice to investigate what impact GPR35 has on the transcriptome. We observed that the mRNA encoding the Na^+^-dependent amino acid transporter SLC6A14 was the top downregulated gene (apart from *Gpr35* itself) in *Gpr35*^−/−^ small intestine (Fig. 3C and Supplementary Data 2). Gene enrichment analysis (+/− using gene ontology sets) of the entire transcriptome revealed ‘Regulation of Voltage Gated Calcium Channels’ as amongst the most upregulated pathway in wildtype mice, and ‘Positive Regulation of Mitochondrial Membrane Potential’ as one of the most upregulated pathway in *Gpr35*^−/−^ intestine. Such changes would be consistent with changes in ion levels within the cells (Fig. 3D). RNAseq also revealed that the Na^+^-dependent amino acid transporter, SLC6A14, is significantly downregulated in *Gpr35*^*–/–*^ compared to *Gpr35*^+/+^ ileal tissue (Fig. 3E). SLC6A14 is highly upregulated in ulcerative colitis and also in solid tumours including breast, colon, liver and pancreatic tumours^46,47^. Energised by the plasma membrane potential, this concentrative transporter imports one amino acid molecule into the cell in symport with 2 Na^+^ and 1 Cl^−^ ^48^. *SLC6A14* mRNA expression was also significantly lower in *GPR35*- compared to control-silenced SW480 cells (Fig. 3F). Upon addition of 50 mM NaCl to the culture medium, SLC6A14 expression increased in *control* but not in *GPR35*-silenced cells (Fig. 3F). We therefore measured [^13^C_5_] glutamine uptake in *SLC6A14* – silenced cells. Like in previous experiments, substantially less [^13^C_5_] glutamine was detected in cell lysates of *GPR35* silenced cells. Silencing control-silenced cells with *SLC6A14* siRNA led to reduced glutamine content but had no effect in already *GPR35*-silenced cells (Fig. 3G).

The 108 M GPR35 risk variant has been shown to increase Na^+^/K^+^-ATPase pump function which may result in increased membrane potential allowing for increased amino acid transport. We therefore measured glutamine levels in cell lysates of KOLF2 iPS cells carrying the 108 M variant and compared these to 108 T cells and found significantly more glutamine in cell lysates from 108 M cells (Fig. 3H).

### Loss of GPR35 leads to impaired proliferation due to sodium overload

We have shown that GPR35 promotes intestinal tumour growth and the turn-over of the intestinal epithelium by promoting Na^+^/K^+^-ATPase-dependent Src kinase activation^17^. Osmotic stress is known to reduce proliferation and cell cycle dynamics in tumour cells^49^. We therefore wondered whether changes in osmotic pressure would contribute to growth inhibition in the absence and presence of GPR35.

**Fig. 4:**
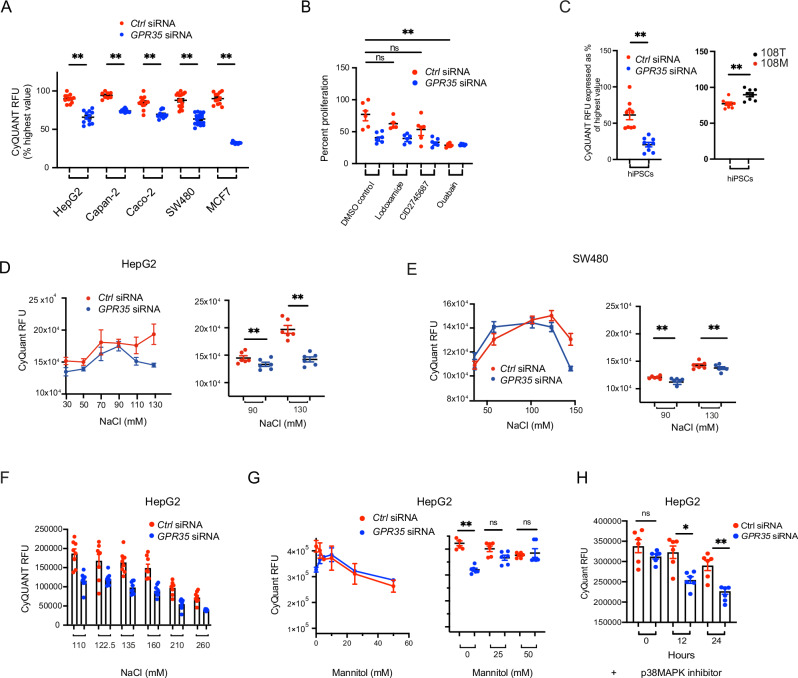
GPR35 regulates Na^+^-dependent proliferation. **A** SiRNA knock-down of *GPR35* in several human cancer cell lines. Proliferation was measured fluorometrically by Cyquant and is shown as percent relative fluorescence units (RFU). *N* = 8 –18 per cell line in *control* and *GPR35* silenced cells. **B** Canonical agonist lodoxamide, antagonist CID2745687 or ouabain added to *control* or *GPR35* silenced HepG2. Proliferation was measured fluorometrically by Cyquant and shown as RFU. *N* = 6 **C** Left panel: Proliferation in *GPR35* silenced hiPSC and control silenced cells. Right panel: Proliferation of hiPSC carrying the risk 108 M variant and cells carrying the non-risk 108 T variant. Proliferation was measured fluorometrically by Cyquant and shown as percent highest RFU. *N* = 9 **D** Proliferation of *control* or *GPR35* silenced HepG2 cells with different concentrations of NaCl in the growth media, measured fluorometrically by Cyquant and shown as RFU. *N* = 6. **E** Proliferation of *control* or *GPR35* silenced SW480 with different concentrations of NaCl in the growth media. Proliferation was measured fluorometrically by Cyquant and shown as RFU. *N* = 6. **F** Proliferation of HepG2 cells in normo- or hyperosmolality. Proliferation of *control* or *GPR35* silenced HepG2 cells was measured fluorometrically by Cyquant and shown as RFU. *N* = 3. **G** Addition of mannitol (5 –50 mM/L) to DMEM media for 12 h in *control* and *GPR35* silenced HepG2 cells. Proliferation was measured fluorometrically by Cyquant and shown as RFU. *N* = 3. **H** Proliferation of *control* or *GPR35* silenced HepG2 cells in the presence of p38 MAPK inhibitor measured after 12 or 24 h. *N* = 6 All data represented as mean ± s.e.m. Statistical significance was calculated using Mann Whitney *U* after Kruskal Wallis testing. *p < 0.05, **p < 0.01.

Cell proliferation following GPR35 knockdown was significantly reduced across a range of cancer cell lines representing intestinal (Caco-2 and SW480), liver (HepG2), pancreatic (Capan-2) and breast (MCF-7) epithelial cells (Fig. 4A). To investigate whether canonical signalling was involved in GPR35-promoted growth, we added GPR35 agonists or antagonists to SW480 cells. These did not affect proliferation (Fig. 4B). The Na^+^/K^+^ATPase inhibitor ouabain, however, reduced proliferation in *control*-silenced cells substantially, down to the level observed in *GPR35*-silenced cells where it had no additional effect (Fig. 4B). The human iPSC line KOLF-2 also exhibited a marked reduction in proliferation following *GPR35* knock-down (Fig. 4C). Moreover, KOLF2 cells carrying the 108 M risk variant proliferated more than those with 108 T (Fig. 4C).

In order to test if the growth phenotype is influenced by intracellular Na^+^ levels, we cultured HepG2 and SW480 cells in cell culture media containing low to physiological levels of total NaCl (30 mM – 130 mM) (Fig. 4D, E). At physiological Na^+^ concentrations of 110–130 mM, *GPR35* silenced cells proliferated significantly less compared to *control* silenced cells. Growth medium containing sub-physiological Na^+^ levels (90–100 mM) led to an increase in proliferation in *GPR35*- silenced cells (Fig. 4D, E). These data suggest that proliferation in *GPR35*- silenced cells under physiological Na^+^ concentrations may be inhibited due to increased intracellular Na^+^ resulting in osmotic stress.

The tumour microenvironment is known to be hypersaline^50^ and cancer cells must therefore adapt whilst maximising sodium-dependent uptake of nutrients. To test how HepG2 cells adapt their growth to high salt, we next exposed these cells to supra-physiological concentrations of Na^+^ in the growth media (Fig. 4F). High salt reduced proliferation in *control* silenced cells in a dose dependent manner, whereas the proliferation rate was more stable in *GPR35* silenced cells up to concentrations of 210 mM. Only concentrations of 260 mM Na^+^ resulted in a substantial further reduction in proliferation (Fig. 4F). Importantly, an equimolar addition of mannitol to the media did not induce similar effects, thus suggesting Na^+^ plays a direct effect on proliferation here (Fig. 4G). Inhibition of p38MAPK worsened the proliferative defect in GPR35 silenced cells suggesting that p38MAPK appropriately engaged a compensatory response to osmotic stress (Fig. 4H).

### High salt diet dampens intestinal regeneration

**Fig. 5:**
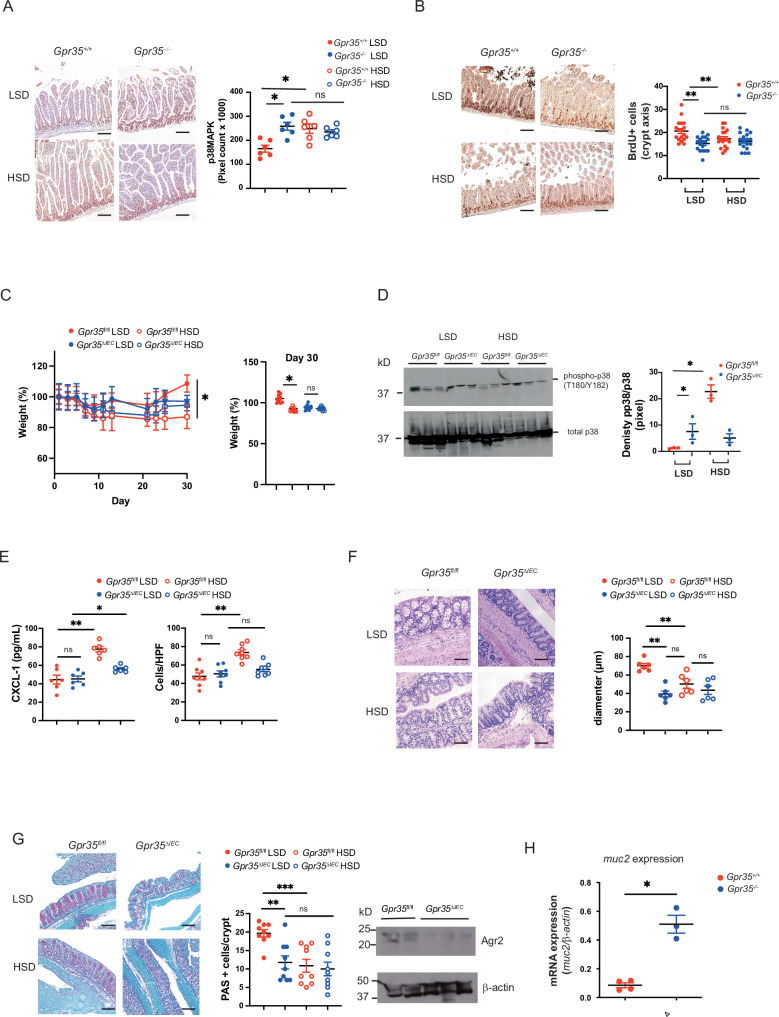
High salt diet induces inflammation and morphological changes in *Gpr35*^*+/+*^ mice. **A** p38 MAPK phosphorylation in *Gpr35*^*+/+*^ and *Gpr35*^−/−^ mice after 7 days of either HSD or LSD. *N* = 7 mice per genotype per diet (*n* = 28) Left panel: representative immune-histochemistry. Right panel: phosphor p38MAPK semiquantitative analysis using Image J. **B** Intestinal epithelial cell proliferation after 7 days of HSD measured as BrdU intake. *N* = 7 mice per genotype per diet (*n* = 28) Left panel: representative immune-histochemistry. Right panel: BrdU positive cells per crypt. **C** Weights of *Gpr35*^*fl/fl*^ and *Gpr35*^*ΔIEC*^ mice under HSD or LSD with 2 additional DSS cycles (2.5% for 4 days). Right panel: weight curve over 30 days. Left panel: weights on day 8 and day 30 of the experiment. *N* = 7 to 11 mice per genotype per diet. **D** Western blot analysis of p38 MAPK phosphorylation in *Gpr35*^*fl/fl*^ and *Gpr35*^*ΔIEC*^ mice under HSD or LSD. Densitometry shown as ratio of phosphorylated p38 MAPK to total p38 MAPK. *N* = 3. **E** Left panel: CXCL1 levels in intestinal biopsies of *Gpr35*^*fl/fl*^ and *Gpr35*^*ΔIEC*^ mice under HSD or LSD measured by ELISA after protein extraction. *N* = 6. Right panel: Infilterating inflammatory cells per high power field (HPF) in *Gpr35*^*fl/fl*^ and *Gpr35*^*ΔIEC*^ mice under HSD or LSD. **F** Goblet cells morphology in *Gpr35*^*fl/fl*^ and *Gpr35*^*ΔIEC*^ mice under HSD or LSD. Left panel: representative H&E stain. Right panel: diameter of goblet cells in µm. *N* = 6. **G** Periodic acid shift (PAS) stain of Goblet cells in *Gpr35*^*fl/fl*^ and *Gpr35*^*ΔIEC*^ mice under HSD or LSD. *N* = 9 per genotype and diet. **H** Expression levels of mucin-2 mRNA in *Gpr35*^*fl/fl*^ and *Gpr35*^*ΔIEC*^ mice. *N* = 3-4. All data represented as mean ± s.e.m. Statistical significance was calculated using Mann Whitney *U* after Kruskal Wallis testing. *p < 0.05, **p < 0.01, scale bars in (**A**–**G**): 100 µm.

High salt diet (HSD) exacerbates colitis in the *Il10*^*–/–*^ colitis model by activating p38 MAPK and SGK1 in dendritic cells. This results in increased pro-inflammatory cytokine expression and p38 MAPK activation in the intestinal epithelium^51,52^. Intestinal liquid in IBD patients has an increased osmolality compared to intestinal fluids of healthy controls, and epithelial cells may therefore be exposed to osmotic stress^8,53^. To investigate GPR35’s role in a high salt diet (HSD), we challenged *Gpr35*^*–/–*^ mice with 2.5 – 3% NaCl in their food for 2 weeks. The intestinal epithelium of *Gpr35*^+/+^ mice exposed to a high compared to a low salt diet exhibited markedly increased p38 MAPK phosphorylation (Fig. 5A). *Gpr35*^–/–^ mice, in contrast, already exhibited higher p38 MAPK phosphorylation on a low salt diet (LSD), and the HSD did not increase this further (Fig. 5A). Importantly, HSD resulted in reduced intestinal proliferation and regeneration in *Gpr35*^+/+^ mice (24 h BrdU pulse after 2 weeks of HSD versus LSD) (Fig. 5B). This reduction in proliferation was comparable to the reduced epithelial proliferation in *Gpr35*^–/–^ mice under LSD, where the HSD did not cause any further reduction in proliferation (Fig. 5B). This suggested that osmotic stress in the intestinal epithelium, caused by either a high salt diet or GPR35 deficiency, prompted a reduction in its regenerative capacity. We have previously described decreased phosphorylation of Na^+^/K^+^-ATPase-dependent Src kinase. This, together with osmotic stress may explain the substantially reduced proliferation observed in the absence of GPR35^17^.

Next, we challenged intestinal epithelial-specific *Gpr35*^ΔIEC^ mice with two cycles of dextran sodium sulphate (DSS) to induce intestinal inflammation, whilst feeding them a HSD or LSD. Weight loss was higher in the *Gpr35*^fl/fl^ HSD group compared to the *Gpr35*^fl/fl^ LSD (Fig. 5C). The weight loss in *Gpr35*^ΔIEC^ mice was not different between LSD and HSD groups (Fig. 5C). Western blots for p38 MAPK phosphorylation in intestinal tissue lysates revealed increased phospho-p38 MAPK compared to total p38 MAPK in *Gpr35*^ΔIEC^ mice on a LSD. Interestingly, this ratio increased substantially in *Gpr35*^fl/fl^ mice on a HSD, whereas the ratio decreased when *Gpr35*^ΔIEC^ mice were on a HSD (Fig. 5D and Supplementary Figure). Levels of the neutrophil chemokine CXCL1 in *Gpr35*^fl/fl^ mice were markedly higher on a HSD compared to a LSD, whilst such increase was not observed in *Gpr35*^ΔIEC^ mice (Fig. 5E). Increased inflammation on HSD was also reflected in the number of infiltrating white blood cells (WBC). Numbers were significantly higher in *Gpr35*^*fl/fl*^ mice compared to their *Gpr35*^*ΔIEC*^ littermates on LSD (Fig. 5E). WBC numbers increased further on a HSD in *Gpr35*^fl/fl^, the increase of recruited WBC in *Gpr35*^*ΔIEC*^ mice was not significant (Fig. 5E).

We and others^54^ observed a profound impact of GPR35 on goblet cells. Goblet cells play a critical role in mucin secretion, prevention of intestinal inflammation and antimicrobial defence^55,56^. Goblet cells of wild-type (*Gpr35*^fl/fl^) mice on a HSD were markedly smaller (Fig. 5F) and numbers were reduced (Fig. 5G) compared to mice in the LSD group. Goblet cells in *Gpr35*^ΔIEC^ mice are already smaller and their numbers reduced under normal LSD conditions, and HSD had no additional impact (Fig. 5F, G and Supplementary Figure). *Muc2* mRNA levels encoding for Mucin-2, the major product of goblet cells, were indeed higher in colonic tissue from *Gpr35*^–/–^ mice compared to *Gpr35*^+/+^ mice (Fig. 5H), pointing toward a compensatory increase. Altogether these data suggest that a diet high in salt leads to increased osmotic stress in wildtype mice leading to decreased epithelial proliferation, increased inflammation and changes in goblet cell morphology. Osmotic stress is already high on LSD in mice with germline or intestinal epithelial-specific GPR35 deficiency, leading to increased p38 MAPK phosphorylation decreased proliferation and decreased Goblet cell size. HSD does not have a substantial further impact on mice lacking GPR35.

## Discussion

Here we report that GPR35 exerts critical control over cellular and organismal Na^+^ and K^+^ homoeostasis, with consequences for cell size, osmotic pressure, and nutrient uptake. Maintaining a gradient between ‘inside’ and ‘outside’ is one of the most fundamental processes of, and prerequisite for, life – from simplest unicellular to complex multi-cellular organisms. The Na^+^/K^+^-ATPase has the central role in maintaining a cell’s electrochemical gradient, at the expense of a substantial proportion of total chemical energy regenerated. It is surprising that a GPCR, unaffected by signalling agonism and antagonism, but by direct interaction with the Na^+^/K^+^-ATPase promoting pump function, would have such pervasive impact.

At cellular level, we observed substantial shape changes and size increases when GPR35 was absent, alongside perturbation in their Na^+^ and K^+^ content and evidence of osmotic stress. Mutant iPS cells expressing the disease risk GPR35-108M, when compared to the −108T variant, exhibited the mirror image of observations in GPR35 silenced or gene-deleted cells, corroborating that −108M is hypermorphic^17^. Strong ‘remote’ consequences of GPR35 deficiency in the intestinal epithelium, i.e. reduced Na^+^ excretion in urine and lower K^+^ serum levels, demonstrate that the very high expression of GPR35 within the intestinal epithelium has profound organismal consequences on ion homoeostasis. The basolateral Na^+^/K^+^-ATPase is the major contributor to high intracellular K^+^ and low intracellular Na^+^ in intestinal epithelial cells^57^. Nutrient (e.g. glutamine and many other amino acids) and Na^+^ uptake occurs on the brush border or apical membrane of intestinal epithelial cells. Cardiotonic steroids indeed can inhibit Na^+^/K^+^-ATPase-dependent amino acid uptake into intestinal epithelial cells^58^. This Na^+^ uptake stimulates the Na^+^/K^+^-ATPase pump on the basolateral membrane to extrude Na^+^ into the subcellular space^59^, a process quantitatively controlled by GPR35. Indeed, the first reported spontaneous phenotype of mice with germline deletion of *Gpr35* was arterial hypertension, pointing to perturbed volume control^60^.

We show that GPR35-deficient cells exhibit features of osmotic stress, even in the context of low salt exposure. Osmo-stress is elicited in wild-type cells by exposure to high salt concentration, either via a high salt diet in vivo, or by elevating salt concentrations in cell culture. Consistent with a major role in osmo-homoeostasis, GPR35 expression is controlled by the osmotic pressure- and NaCl-sensitive transcription factor NFAT5. In renal medullary cells, NFAT5 is a regulator of the expression of osmo-protective genes^61^. NFAT5 is expressed in the intestinal tract and haploinsufficiency has been associated with enterocolopathy^62,63^. Although the main focus in those reports was on T cell biology, NFAT5 is widely expressed, including in the intestinal epithelium.

High salt content, a key feature of Westernised diets, has been associated with autoimmune pathologies, such as inflammatory bowel disease^10^. High salt diet can, for example, promote Th17 cell differentiation and autoimmune pathologies associated with them^13,14^. Lower levels of GPC in inflamed biopsies from ulcerative colitis patients indicate presence of osmotic stress in the mucosa, albeit it necessarily remains unclear whether this is a primary or compensatory feature. GPR35 protects the intestinal epithelium against experimental colitis induced by dextran sodium sulphate^64,65^. Melhem et al. have reported a reduction in goblet cell numbers^66^, consistent with our observations. Challenged with a high salt diet, goblet cell numbers and morphology changed in wildtype mice, but did not further deteriorate those in *Gpr35*^*ΔIEC*^ mice. This suggests that osmotic stress and salt overload causes altered goblet cell numbers and morphology, which have a critical role in the microbiome—host interface^55^.

Why does a *receptor* control Na^+^/K^+^-ATPase activity, and what does it respond to? As noted above, none of the GPR35 pharmacological agonists and antagonists yet tested impacted on pump activity. It is tempting to speculate that endogenous ligand(s) exist that may directly impact on pump activity whilst not eliciting canonical G protein signalling. The notion that the 108 M variant enhances pump function represents a hint that such direct control exists. Understanding the biophysical basis of this control will be important.

## Materials and methods

### Materials

All key resources are listed in supplementary data 1.

### Mice

Age- and gender-matched 6- –12 week-old mice were used for all experiments. Maintenance and breeding under specific pathogen-free conditions was performed the Anne McLaren Animal Building, University of Cambridge. The study was approved by the UK Home Office (PP3638130) and local ethics (University Biomedical Services, University of Cambridge, https://www.ubs.admin.cam.ac.uk) has been granted for all experimental procedures. Gpr35 knock-out mice were obtained from the KOMP repository (clone ID 646854). Conditional knock-out mice were generated by outbreeding the FlpO and then breeding in Villin-Cre as described^17^. Both strains littered at Mendelian ratios and developed normally without any spontaneous disease. We have complied with all relevant ethical regulations for animal use and adhered strictly to the ARRIVE guidelines as required by the University of Cambridge and Wellcome Trust.

We used an extensive set of in vitro reductionistic experiments however relied on mouse experimentation to study GPR35 in a relevant physiological context. To avoid cage effects and to have the appropriate littermate wild-type control mice available mice were bred heterozygously for the global and conditional mice. We fully embraced the 3Rs, and the entire project is arranged along these lines trying to keep animal numbers low. No animals were excluded.

### High salt diet

Six to twelve week old *Gpr35*^−/−^ and wildtype littermates were assigned to either receive a high salt diet (HSD) (2.5–3% NaCl) or were on a control diet (LSD) containing 0.25 – 0.3% NaCl for 2 weeks. Mice were injected with BrdU 24 h before the termination of the experiment and intestines were collected for histolog which were analysed in a blinded manner. Performance of power calculations led us to a number of 6 animals per group to detect a pharmacological effect size of >2.0 with a statistical power of >0.85.

To minimise confounders the diet was exchanged during morning hours when food intake is lowest. BrDU was injected by mixing the allocated diet or genotype.

In a second set of experiments intestinal epithelial cell specific Gpr35 knock-out (*Gpr35*^*ΔIEC*^) and wildtype mice (*Gpr35*^*fl/fl*^) (littermates) were on a LSD or HSD (randomly assigned) and subjected to 2 cycles of Dextran Sulfate Sodium (DSS) salt, (MP Biomedicals, MW 36–50 kDa), 2.5% in autoclaved drinking water. Weights were taken every other day for 50 days. To avoid cage effects beddings were swapped throughout the experiment. The incidence of DSS induced colitis ranges from 0.25 to 0.8. Power analysis (available at www.stat.ubc.ca) based on chi- squared statistics, with power value = 0.85, significance level: *p* < 0.05, 2-sided indicated *n* = 10 per group. Groups were assigned randomly, *Gpr35*^*fl/fl*^ on a LSD or HSD, *Gpr35*^*ΔIEC*^ on either a LSD or HSD.

### siRNA transfection

SMARTpool: ON-TARGETplus human GPR35 siRNA (Horizon #L-005567), human NFAT5 (Horizon ## L-009618-00), human SLC6A14 (Thermo Fisher Silencer #AM16708) and ON-TARGETplus Non-targeting siRNA (Horizon #D-001810) were purchased from Horizon Discovery or Thermo Fisher and diluted to a 20 μM stock. siRNA silencing was performed using a reverse transfection protocol. siRNA was combined with lipofectamine RNAiMAX in OptiMem medium according to manufacturer’s instructions before adding 10uL to each well of a 96 well plate. Cells were the plated on top of this at a density of 5,000 cells per well in 90uL normal growth medium (DMEM + 10% FBS). The final concentration of siRNA was 10 nM

### Cell size measurement

Cell size was measured using FACS forward scatter (FSC) as a surrogate marker of cell volume. Tumour cells or murine bone marrow derived macrophages were trypsinised and resuspended in HBSS with magnesium, calcium chloride and Hepes 10 mM. The Attune NxT flow cytometer (Thermo Fisher) was then used to measure baseline FSC. Cell size was assessed using the Invitrogen Flow Cytometry Calibration kit (Thermo Fisher) with bead sizes ranging from 4 µm to 15 µm.

### Cell proliferation measurement

Cell proliferation was measured using the CyQUANT cell proliferation assay (Thermo Fisher). Cells were reverse transfected as above at a density of 5000 cells per well in 96 well black chimney clear bottom plates. 72 h post transfection the culture medium was replaced with the CyQUANT dyes as per manufacturer’s instructions. After 1 h incubation the cell density was measured using in a 7 × 7 grid per well using the CLARIOstar Plus plate reader (BMG Labtech) at 483/530 nm excitation/emission wavelengths. Fluorescence intensity using this technique is linearly dependent on cell number.

### Western blotting

Cells were lysed in RIPA buffer (50 mM Tris, pH 7.4, 150 mM NaCl, 1% Nonidet P-40, 0.5% sodium deoxycholate, and 0.1% SDS). For nuclear extracts cytoplasm was separated from nuclei and nuclei extracts were isolated by using hypertonic lysis. Protein content was then measured using the BCA assay (Pierce) and equal amounts of lysates mixed with Laemmli buffer. Samples were then subjected to SDS-PAGE. After blotting onto Hybond-P polyvinylidene fluoride membranes (GE Healthcare) blots were blocked with 5% milk in TBS-T, and primary antibody, in 5% BSA in TBS-T was added at 4 °C overnight. The protein was then detected by using a HRP-conjugated secondary antibody, and visualised with LumiGLO (Cell Signalling Technology).

### Crispr/Cas9 editing of human iPSC line

The rs3749171 risk variant (a single base substitution (C > T)) was introduced in Kolf2 cells by Cas9-crRNA-tracrRNA. The sequences for the RNA oligonucleotides were 5′- CCTGGTCACGGCCATCGCCG -3′ or 5′ CACATAGCGGTCCACGGCGA 3′ and described in Schneditz et al. {Schneditz, 2019 #152}.

### LC-MS Metabolomics

HepG2 cells were reverse transfected with siRNA at a density of 250,000 cells/well in 6 well plates. After 48 h incubation under normal culture conditions, cells were washed twice with 2 mL/well phosphate free buffer (162 mM ammonium acetate adjusted to pH 7.4 with ammonia) before adding 1 mL/well ice cold 4:1 methanol:water. A cell scraper was used to lift the cells and the suspension was transferred to clean microcentrifuge tubes using a p1000 pipette.

Human biopsies were obtained after gaining consent from patients during routine endoscopies (Cambridge University Hospitals NHS Foundation Trust and the University of Cambridge, IRAS: 298628, non identifiable patient samples) and snap frozen for further evaluation.

The cell or human samples were then freeze dried in a centrifugal evaporator. Folch extraction was then performed by resuspending each dry pellet in 666 μL chloroform followed by the addition of 333 μL methanol and 400 μL water. This was then vortexed thoroughly and sonicated in a water bath sonicator. A clean stainless steel metal ball was then added to each tube and this was pulsed in a homogeniser at 20 Hz for 3 × 30 s pulses. The tube was then centrifuged at 17,000 G for 5 min creating an upper aqueous phase and lower lipid phase. The upper aqueous phase was transferred to a microcentrifuge tube. The lipid fraction was transferred to a glass vial. Samples were then dried again using a centrifugal evaporator. The dried aqueous fraction was then reconstituted in 50 μL of 10 mM ammonium acetate containing an internal standard mixture of 4uM 13 C and 15 N labelled AMP and ATP, 10uM 13 C succinate and 1 in 5000 diluted 13 C, 15 N cell free amino acid mixture (Sigma #767964). Samples were then analysed using a “Q Exactive Orbitrap Mass Spectrometer (Thermo Fisher) and data was normalised to total ion content and compounds identified using Compound Discoverer software.

### Intracellular ion concentration measurement

Intracellular sodium was measured using the ratiometric dye SBFI, AM (Thermo Fisher, Cat S1264). Intracellular potassium was measured using the ratiometric dye PBFI AM (Thermo P1267MP). 50ug of dye was resuspended in 4.4 uL DMSO. This was then mixed in a 1:1 ratio with Pluronic F-127 (Thermo Cat P3000MP). 1uL of dye mix was diluted in 1 mL cell culture medium (DMEM + 10% FBS). Cells were reverse transfected as above and plated at a density of 10,000 cells/well in a clear bottom black chimney 96 well plates. 48 h post transfection the culture medium was replaced with the dye mix and cells were incubated for 3 h in a 37 °C, 5% CO2 incubator. The dye mix was then replaced with normal culture medium and the ratiometric fluorescence measured using a CLARIOstar Plus plate reader (BMG Labtech) at 340/500 & 380/500 nm excitation/emission wavelengths. Intracellular ion concentration was expressed as a ratio of fluorescence intensity at 340/380 excitations to correct for background variation. For ligand experiments the ligand of interest was added 30 min prior to measurement in a concentrated form to achieve the desired final concentration in the dye mix.

### Potassium flux measurement

The FluxOR II Green Potassium Ion Channel Assay (Thermo F20016) was used according to manufacturer’s instructions. Briefly, cells were reverse transfected as above and plated at a density of 10,000 cells/well in a clear bottom black chimney 96 well plates. 48 h post transfection cells were loaded with thallium sensitive dye in loading buffer with probenecid for 60 min at room temperature. The loading buffer was then replaced with assay buffer. The CLARIOstar Plus plate reader (BMG Labtech) was then used to measure fluorescence intensity at 480/530 excitation/emission every 2 s prior to and following injection of thallium using the dispensing function of the plate reader. To test voltage gated potassium channel activity, potassium sulfate was added to the thallium injection to elevate extracellular potassium levels to 10 mM.

### Glutamine assay

Cells were reverse transfected as above in 96 well plates. At each time point 5uL of the cell culture medium was removed from a set of wells and transferred to microcentrifuge tubes containing 95uL cold PBS. These were centrifuged to remove cells and debris before being frozen at -80 °C until all time points were collected. The samples were then thawed on ice and the Glutamine/Glutamate GLO assay (Promega J8021) was used to measure the concentration of glutamine in each sample according to manufacturer’s instructions. Luminescence was measured using the CLARIOstar Plus plate reader (BMG Labtech).

### Immunohistochemistry

Paraffin-embedded samples were then deparaffinized in xylene and dehydrated with ethanol. Antigen retrieval was performed by boiling the slides for 12 min in citrate unmasking solution (Cell Signalling, #14746). Cells positive for BrdU were detected using a BrdU In-Situ Detection Kit (BD Pharmingen, 550803). Proliferation was revealed as BrdU+ cells along the crypt-villus axis. phospho-p38MAPK was detected with a primary phospho-p38 MAPK (Thr180/Tyr182) antibody (Cell Signalling Technology, D3F9). Antigen retrieval was performed by boiling the slides for 12 min in citrate unmasking solution (Cell Signalling Technology, #14746). After incubation in 3% hydrogen peroxide slides were washed and incubated with 1X Animal-Free Blocking Solution (Cell Signalling Technology, #15019) for 1 h. The primary antibody was then added to the slides and incubated overnight at 4 °C. After washing SignalStain® Boost IHC Detection Reagents (Cell Signalling Technology, HRP, Rabbit #8114) was added and incubated for 30 min in a humidified staining box. After washing SignalStain® DAB Substrate Kit (Cell Signalling Technology,#8059) was added and slides were counterstained with hematoxylin (Cell Signalling Technology, #14166). After rinsing slides were dehydrated and mounted with SignalStain® Aqueous Mounting Medium (Cell Signalling Technology, #27290). phospho-p38 MAPK positive cells were counted microscopically.

### Periodic Acid Schiff (PAS) staining

Tissue sections were deparaffinised and hydrated with xylene and ethanol. Slides were then immersed in Periodic Acid Solution (SigmaAldrich, #3951-100 ML) for 5 min at room temperature. The slides were then rinsed in several changes of distilled water before incubating in Schiff’s Reagent (Sigma Aldrich, #3952-50 ML) for 15 min at room temperature. After washing slides were counterstained with hematoxylin (Cell Signalling Technology, #14166). After rinsing slides were dehydrated and mounted with SignalStain® Aqueous Mounting Medium (Cell Signalling Technology, #27290). PAS positive cells were then counted microscopically.

### RNAseq

Total RNA was extracted from ileum or bone marrow derived macrophages from *Gpr35*^*+/+*^ *or Gpr35*^−/−^ mice, using RNeasy Mini Kit (QIAGEN, 74104), libraries prepared using the TruSeq stranded mRNA library prep kit, processed and sequenced on the Illumina HiSeq 2000 50-bp single-end reads platform. RNA seq was performed at Norwegian Sequencing Centre. Enrichment analysis was undertaken using GSEA software (UC San Diego and Broad Institute) and ontology gene sets of the Mouse Molecular Signatures Database (MSigDB). Data were downloaded onto the Geoserver (https://geoserver.org).

### Human samples

Patient samples were taken after signed consent forms. Patients were undergoing routine colonoscopy. Ethics were approved by the Cambridge University Hospitals NHS Foundation Trust and the University of Cambridge, IRAS 298628. All ethical regulations relevant to human research participants were followed.

### Human macrophage preparation

Buffy coats were diluted 1:1 in room temperature PBS and layered on top of Lymphoprep® (Pharmacia, Uppsala, Sweden) and centrifuged at 1500 rpm for 25 min. The interface was then collected and washed three times with cold PBS (10 min of 1600 rpm). Cells were then placed into a 15 cm dish fand left in a humidified incubator at 37 °C in for 90 min. Non adherent cells were washed off afterwards and macrophage media was added (RPMI1640, 2 mM glutamine, 50 U/mL penicillin, 50 µg/mL streptomycin, 50 ng/ml M-CSF, 25 ng/ml interleukin-10, and 10% FBS. Cells were harvested after 5 days.

### Statistics and reproducibility

Statistical analyses were performed using Graphpad Prism 9 or, and as described in LC-MS analysis methods, Compound Discoverer 2.1 (Thermo Scientific). Unless otherwise stated, statistical significance was calculated as appropriate using either unpaired, two-tailed Student’s *t*-test or ordinary one-way ANOVA and Tukey post hoc test as described in the figure legends. Analysis for pooled experiments were performed by using Mann-Whitney test after Kruskal-Wallis testing (*p* < 0.01). All in vivo experiments and image analysis were performed in a blinded manner. Data are represented as mean and standard error of the mean (S.E.M.). *P*-values of <0.05 were considered significant. ns not significant, *p < 0.05, **p < 0.01. Numbers of individual data points are given in the figure legends.

### Reporting summary

Further information on research design is available in the Nature Portfolio Reporting Summary linked to this article.

## Supplementary information

Supplementary Information

Suppl Data 1

Suppl Data 2

Suppl Data 3

Description of Additional Supplementary Materials

Reporting Summary

## Data Availability

All key resources and sequences of primers and siRNAs can be found in Supplementary Data 1. Datasets generated are included in the published article, available within the paper and in its Supplementary Information (Supplementary Data 3 includes all numerical data for figure panels). RNAseq data were downloaded onto the Geoserver (https://www.ncbi.nlm.nih.gov/geo/query/acc.cgi?acc=GSE252926) and raw data are provided in Supplementary Data 2. Metabolomics data will be made available on reasonable request by the authors (nk428@cam.ac.uk). All data are stored on the University of Cambridge’s cloud and on the laboratory’s devices.
