# Supplementary material for: GPR35 prevents osmotic stress induced cell damage
Source: Commun Biol. 2025 Mar 22;8:478. doi: 10.1038/s42003-025-07848-9 (PMC11929815; doi:10.1038/s42003-025-07848-9)
Supplement: Supplementary file 1 — Supplementary Information [file 42003_2025_7848_MOESM1_ESM.pdf]

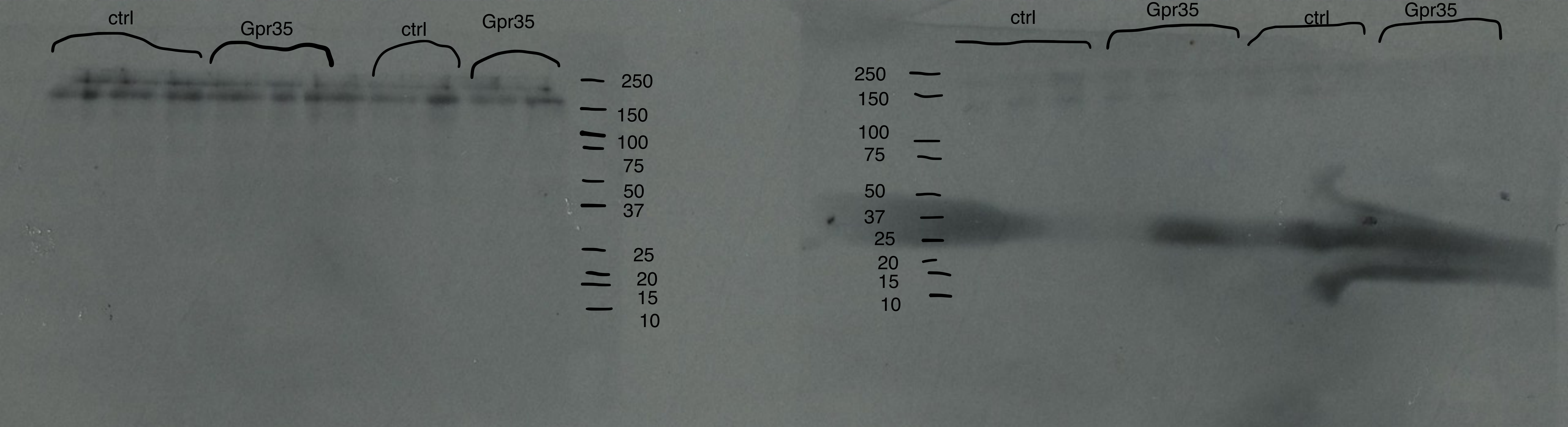
ctrl
Gpr35
ctrl
Gpr35
250
150
100
75
50
37
25
20
15
10
250
150
100
75
50
37
25
20
15
10
ctrl
Gpr35
ctrl
Gpr35

Figure 2H

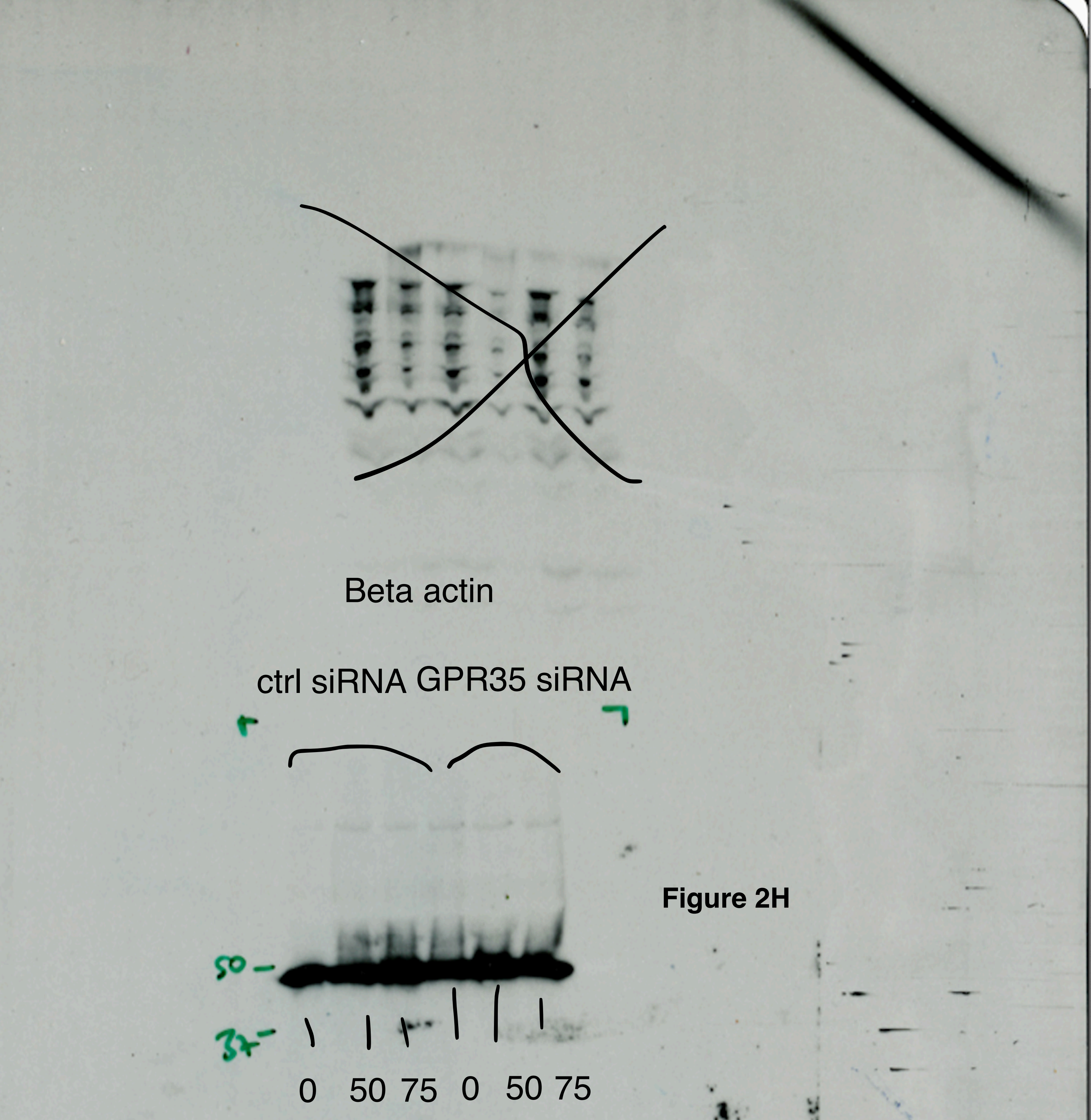

Figure 2H

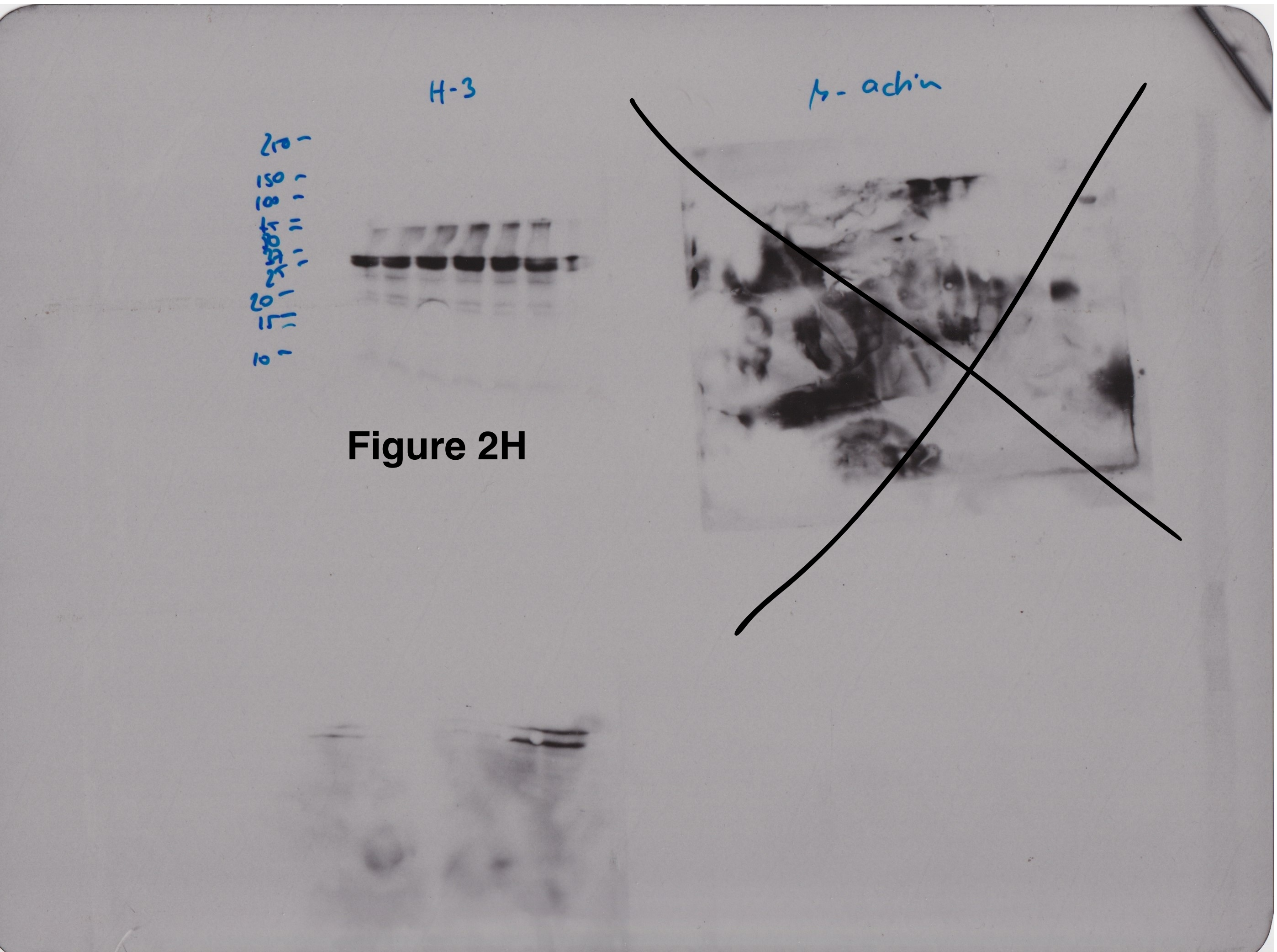

Figure 2H

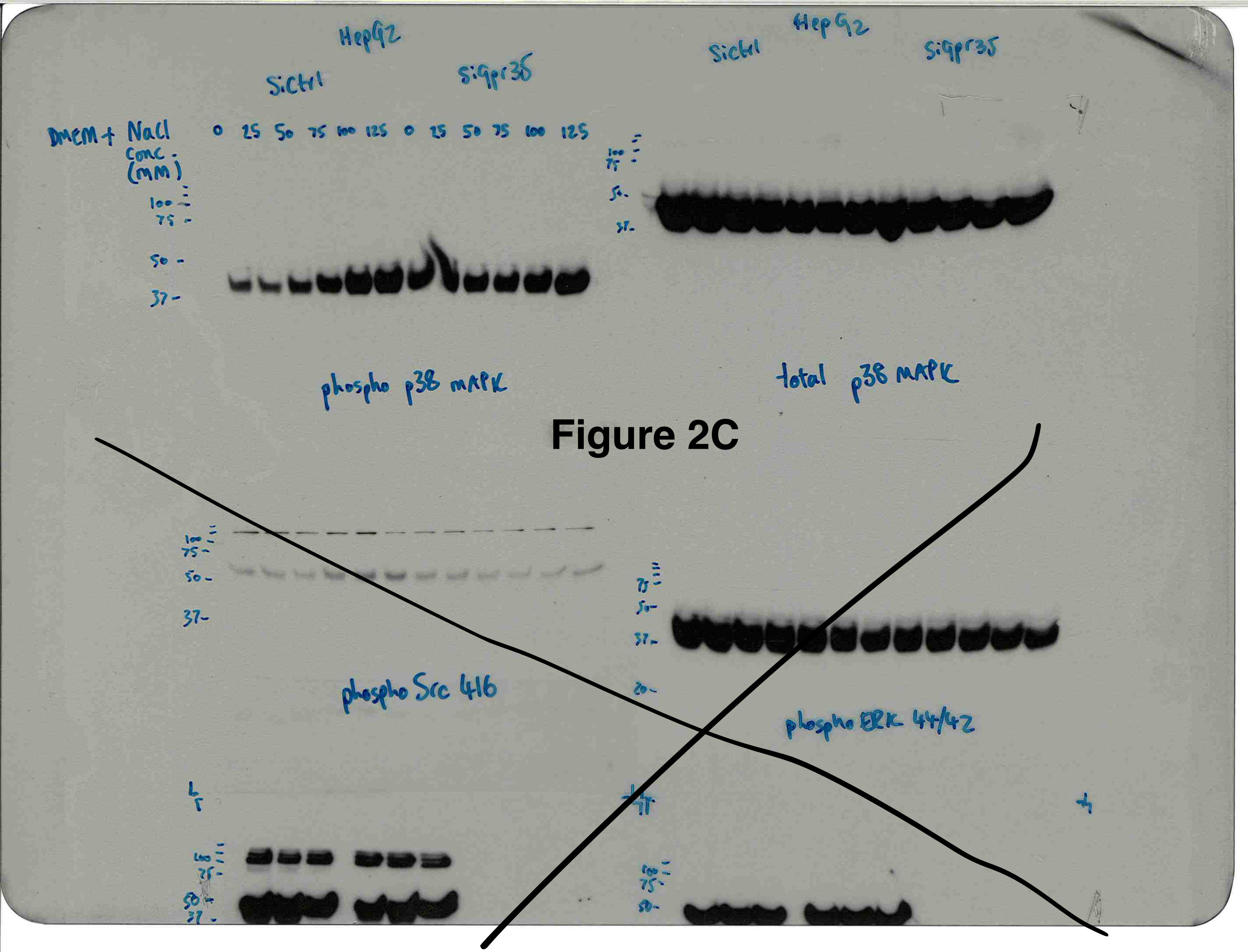
HepG2
SiCtrl
SiGpr35
DMEM + NaCl conc. (mM)
0 25 50 75 100 125 0 25 50 75 100 125
phospho p38 MAPK
total p38 MAPK
phospho Src 416
phospho ERK 44/42

Figure 2C

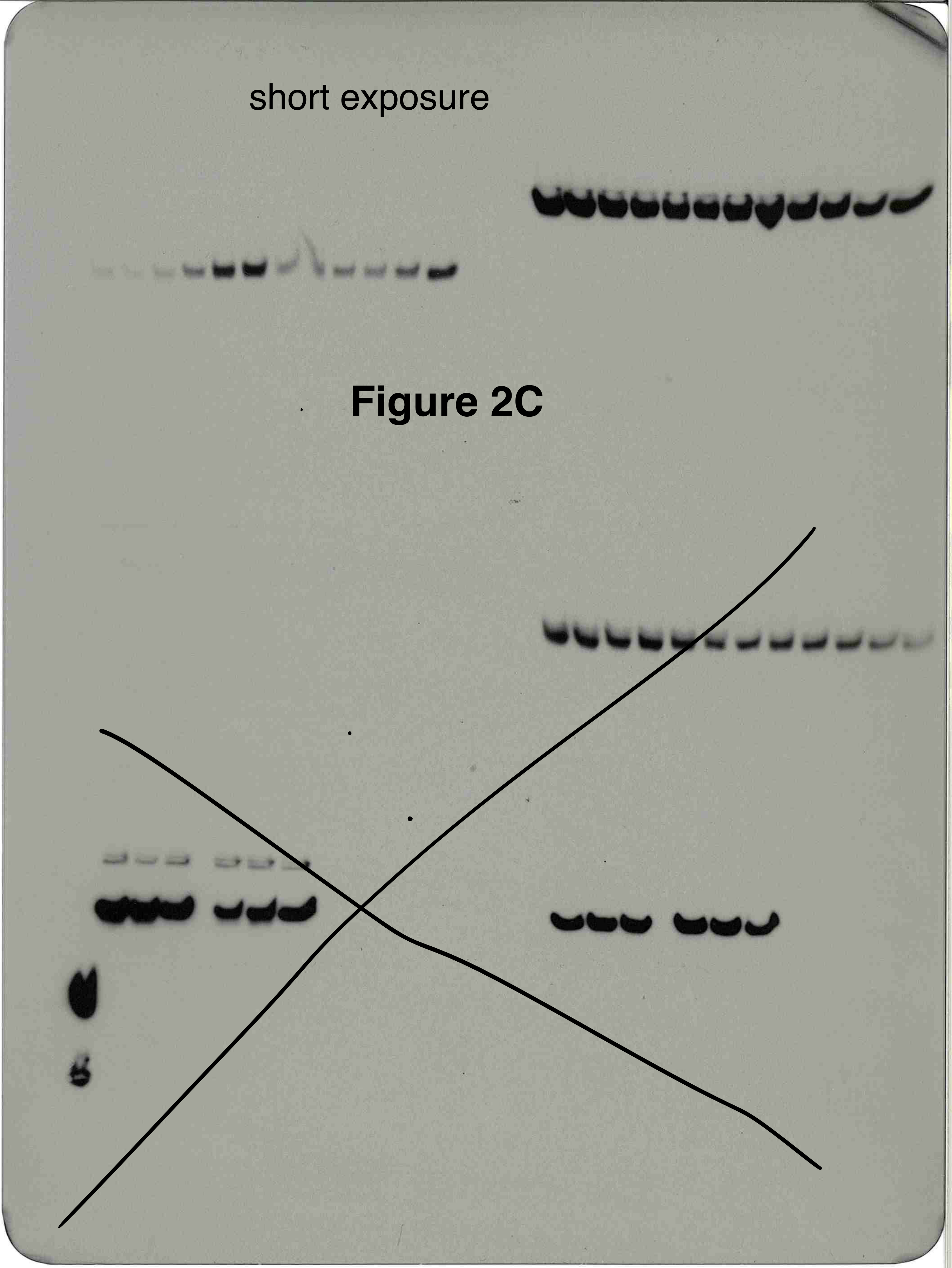

Figure 2C

Figure 5G

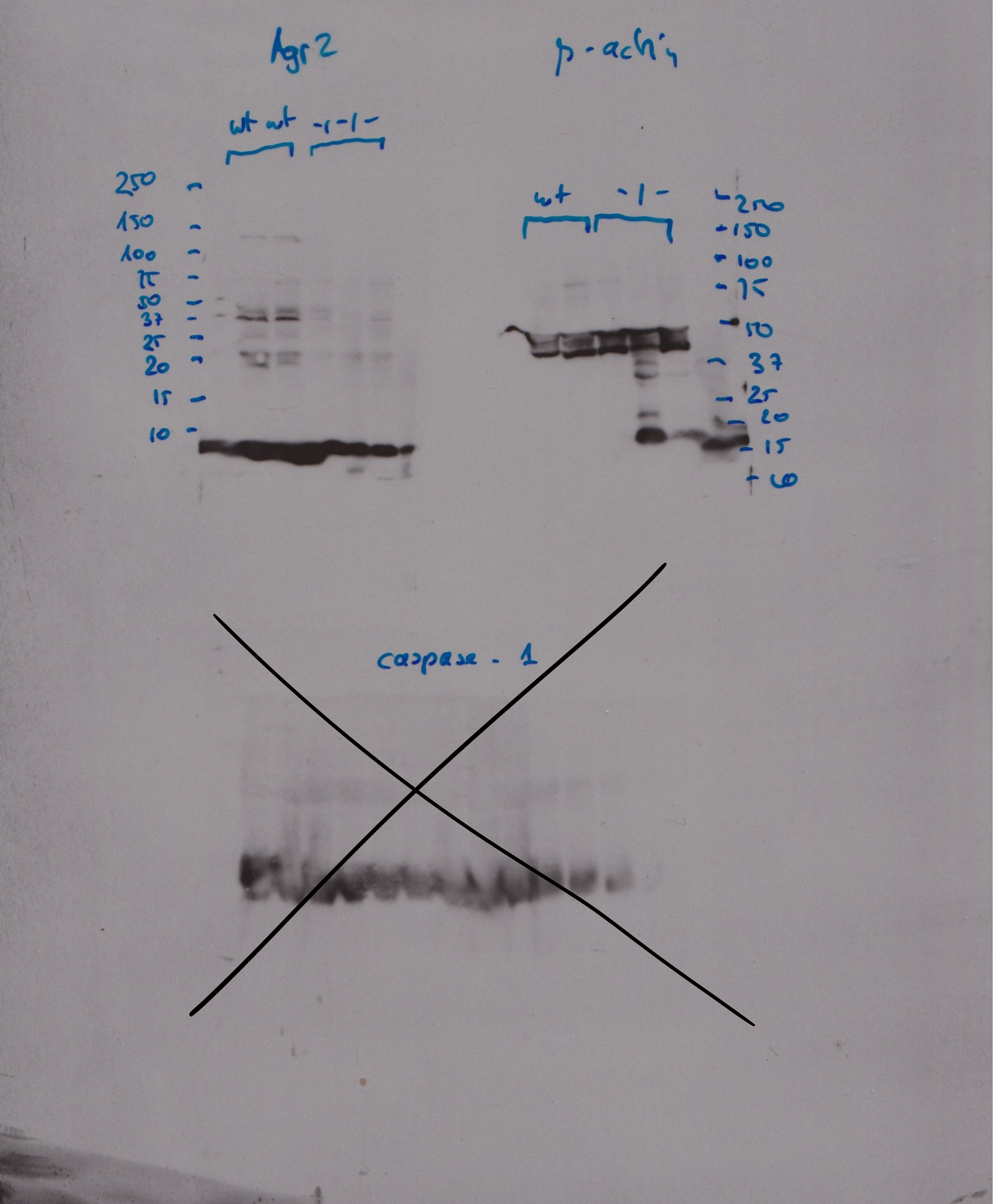
Agr2
wt wt -/-/-
250
150
100
75
50
37
25
20
15
10
β-actin
wt -/-
250
150
100
75
50
37
25
20
15
10
caspase - 1

Figure 5D

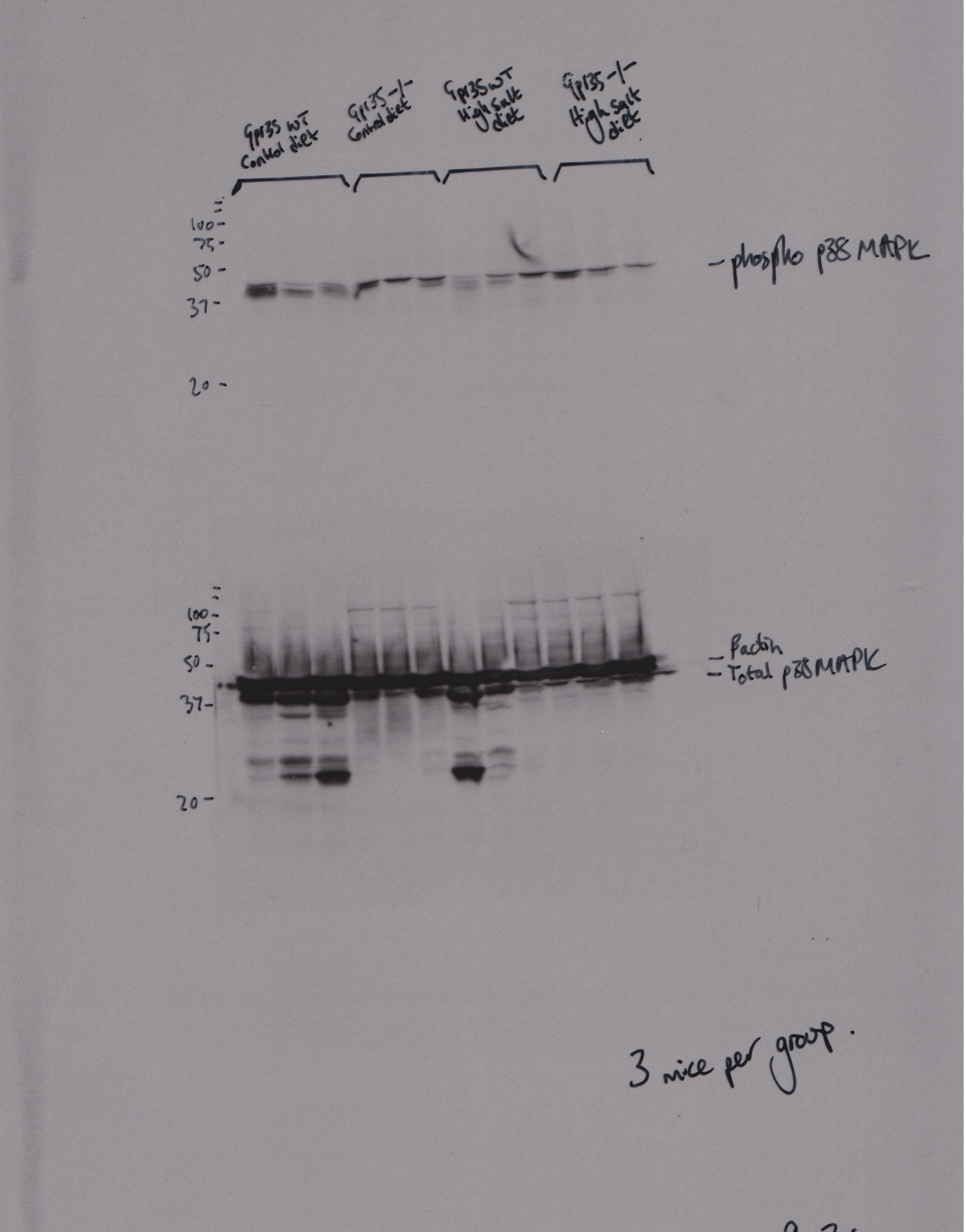

Gpr35 WT Control diet
Gpr35 -/- Control diet
Gpr35 WT High salt diet
Gpr35 -/- High salt diet
100
75
50
37
20
phospho p38 MAPK
100
75
50
37
20
βactin
Total p38MAPK
3 mice per group.
