# Supplementary material for: GPR35 prevents osmotic stress induced cell damage
Source: Commun Biol. 2025 Mar 22;8:478. doi: 10.1038/s42003-025-07848-9 (PMC11929815; doi:10.1038/s42003-025-07848-9)
Supplement: Supplementary file 2 — Suppl Data 1 [file 42003_2025_7848_MOESM2_ESM.pdf]

Key resources table

REAGENT	SOURCE	IDENTIFIER
**Antibodies**		
Phospho-p38 MAPK (Thr180/Tyr182) [D3F9]<br>1:1000 for Western Blotting<br>1:500 for Immunohistochemistry	Cell Signaling Technology	Cat#4511S
Total p38 [D13E1]<br>1:1000 for Western Blotting	Cell Signaling Technology	Cat#8690S
β-actin [13E5]<br>1:1000 for Western Blotting	Cell Signaling Technology	Cat#4970L
NFAT5<br>1:1000 for Western Blotting	Thermo Fisher	Cat#PA-023
Agr2<br>1:1000 for Western Blotting	Cell Signaling Technology	Cat#13062
**Chemicals, peptides, and recombinant proteins**		
Lipofectamine RNAiMAX	Thermo Fisher Scientific	Cat#13778
SBFI	Thermo Fisher Scientific	Cat#S1264
PBFI	Thermo Fisher Scientific	Cat# P1267MP
Pluronic F-127 (Thermo)	Thermo Fisher Scientific	Cat#P3000MP
Ouabain octahydrate	Merck	Cat#O0200000
Lodoxamide	Chemcruz	Cat#sc-489390
CID-2745687	Chemcruz	Cat#sc-362725
ML-145	Bio-techne	Cat#4172
SQ22536	Cayman Chemicals	Cat#13339
KH7	Cayman Chemicals	Cat#CAY13243
pNaKtide	Schneditz et al.	
SB203580 (P38 inhibitor)	Cell Guidance Systems	Cat#SM32
Mannitol	Merck	Cat#M4125
L-Glutamine (200mM)	Thermo Fisher Scientific	Cat#25030081
L-Glutamine-13C5	Merck	Cat#605166
LumiGLO chemiluminescent substrate	Cell Signaling Technology	Cat#7003
**Critical commercial assays**		
FluxOR II Green Potassium Ion Channel Assay	Thermo Fisher Scientific	Cat#F20016
CyQUANT cell proliferation assay	Thermo Fisher Scientific	Cat#C7026
Glutamine/Glutamate GLO assay	Promega	Cat#J8021
**Experimental models: Cell lines**		
HepG2	ATCC	ATCC no. HB-8065<br>RRID:CVCL_0027

SW480	ATCC	ATCC no. CCL-228<br>RRID:CVCL_0546	
Capan-2			ATCC
Caco-2	ATCC	ATCC no.HTB-37<br>RRID:CVCL_0025	
MCF-7	ATCC	ATCC no. HTB-22<br>RRID:CVCL_0031	
KOLF-2 (HPSI0114i-kolf_2-C1)	Wellcome Trust Sanger Institute	hPSCreg: WTSIi018-B-1<br>Cellosaurus: CVCL_9S58	
**Experimental models: Organisms/strains**			
Mouse: Gpr35$^{-/-}$	KOMP repository	Clone ID 646854	
Mouse: Gpr35$^{\Delta}$IEC (Gpr35 fl/fl Villin Cre), Gpr35tm2e(EUCOMM)Wtsi	Conditional knock-out mice were generated by the Wellcome Trust Sanger Institute	MGI:4938582	
**Oligonucleotides**			
GPR35 siRNA<br>Target sequences:<br>1: GGACGGAGACCCGCAUCUA<br>2: GGAGCACCCGGCACAAUUU<br>3: CCAGCAAGCUCUCAGAUGC<br>4: GCAUCUACCUGACCAACAG	Horizon	#L-005567	
Control siRNA<br>Sequences:<br>1: UGGUUUACAUGUCGACUAA<br>2: UGGUUUACAUGUUGUGUGA<br>3: UGGUUUACAUGUUUUCUGA<br>4: UGGUUUACAUGUUUUCCUA	Horizon	ON-TARGETplus, Non-targeting control pool<br># D-001810-10	
NFAT5 siRNA<br>Target sequences:<br>1: CAACAUGCCUGGAAUUCAA<br>2: CAACAUGCCUGGAAUUCAA<br>3: CAGCAGUUAUCUUCAGUUU<br>4: CAGAGUCAGUCCACAGUUU	Horizon	ON-TARGETplus, smartpool<br># L-009618-00	
Slc6a14 siRNA<br>Target sequence<br>ACTGGTCCAAAAAATCGGAT	Thermo Fisher	Silencer<br># AM16708	
qPCR FWD primer: human *GPR35*<br>CCCTTCTCAGACAGCCACTG	Merck Life Science Limited		
qPCR REV primer: human *GPR35*<br>GGTGCCATTCATGGTCCTGC	Merck Life Science Limited		
qPCR FWD primer: human *NFAT5*<br>CCGAAGAGGCACACAGTCTT	Merck Life Science Limited		
qPCR REV primer: human *NFAT5*<br>ACTCCTGCTGGCTGAGTACA	Merck Life Science Limited		
qPCR REV primer: human *SLC6N14*<br>AGG ACC ACA TAG GGG AAA AG	Thermo Fisher	MGH primer bank	
qPCR FWD primer: Murine muc2<br>TCCAGGTCTCGACATTAGCAG	Thermo Fisher	MGH primer bank	
qPCR REV primer: Murine muc2<br>GTGCTGAGAGTTTGCGTGTCT	Thermo Fisher	MGH primer bank	
