# Supplementary material for: GPR35 prevents osmotic stress induced cell damage
Source: Commun Biol. 2025 Mar 22;8:478. doi: 10.1038/s42003-025-07848-9 (PMC11929815; doi:10.1038/s42003-025-07848-9)
Supplement: Supplementary file 5 — Description of Additional Supplementary Materials [file 42003_2025_7848_MOESM5_ESM.pdf]

Description of Additional Supplementary Files

File name: Supplementary Data 1
Description: Key resources

File name: Supplementary Data 2
Description: RNAseq raw data

File name: Supplementary Figures
Description: Western Blots
