# Supplementary material for: GPR35 prevents osmotic stress induced cell damage
Source: Commun Biol. 2025 Mar 22;8:478. doi: 10.1038/s42003-025-07848-9 (PMC11929815; doi:10.1038/s42003-025-07848-9)
Supplement: Supplementary file 6 — Reporting Summary [file 42003_2025_7848_MOESM6_ESM.pdf]

nature portfolio

Corresponding author(s): Nicole C Kaneider

Last updated by author(s): Jan 13, 2025

Reporting Summary

Nature Portfolio wishes to improve the reproducibility of the work that we publish. This form provides structure for consistency and transparency in reporting. For further information on Nature Portfolio policies, see our Editorial Policies and the Editorial Policy Checklist.

Statistics

For all statistical analyses, confirm that the following items are present in the figure legend, table legend, main text, or Methods section.

n/a	Confirmed	
☐	☒	The exact sample size (*n*) for each experimental group/condition, given as a discrete number and unit of measurement
☐	☒	A statement on whether measurements were taken from distinct samples or whether the same sample was measured repeatedly
☐	☒	The statistical test(s) used AND whether they are one- or two-sided<br>*Only common tests should be described solely by name; describe more complex techniques in the Methods section.*
☒	☐	A description of all covariates tested
☒	☐	A description of any assumptions or corrections, such as tests of normality and adjustment for multiple comparisons
☒	☐	A full description of the statistical parameters including central tendency (e.g. means) or other basic estimates (e.g. regression coefficient) AND variation (e.g. standard deviation) or associated estimates of uncertainty (e.g. confidence intervals)
☒	☐	For null hypothesis testing, the test statistic (e.g. *F*, *t*, *r*) with confidence intervals, effect sizes, degrees of freedom and *P* value noted<br>*Give P values as exact values whenever suitable.*
☒	☐	For Bayesian analysis, information on the choice of priors and Markov chain Monte Carlo settings
☒	☐	For hierarchical and complex designs, identification of the appropriate level for tests and full reporting of outcomes
☒	☐	Estimates of effect sizes (e.g. Cohen's *d*, Pearson's *r*), indicating how they were calculated

Our web collection on statistics for biologists contains articles on many of the points above.

Software and code

Policy information about availability of computer code

Data collection	n/a
Data analysis	n/a

For manuscripts utilizing custom algorithms or software that are central to the research but not yet described in published literature, software must be made available to editors and reviewers. We strongly encourage code deposition in a community repository (e.g. GitHub). See the Nature Portfolio guidelines for submitting code & software for further information.

Data

Policy information about availability of data

All manuscripts must include a data availability statement. This statement should provide the following information, where applicable:

- Accession codes, unique identifiers, or web links for publicly available datasets
- A description of any restrictions on data availability
- For clinical datasets or third party data, please ensure that the statement adheres to our policy

n/a

Research involving human participants, their data, or biological material

Policy information about studies with human participants or human data. See also policy information about sex, gender (identity/presentation), and sexual orientation and race, ethnicity and racism.

Reporting on sex and gender	n/a
Reporting on race, ethnicity, or other socially relevant groupings	n/a
Population characteristics	n/a
Recruitment	Patient samples were taken after signed consent forms. Patients were undergoing routine colonoscopy.
Ethics oversight	Ethics were approved by the Cambridge University Hospitals NHS Foundation Trust and the University of Cambridge, IRAS 298628

Note that full information on the approval of the study protocol must also be provided in the manuscript.

Field-specific reporting

Please select the one below that is the best fit for your research. If you are not sure, read the appropriate sections before making your selection.

☒ Life sciences ☐ Behavioural & social sciences ☐ Ecological, evolutionary & environmental sciences

For a reference copy of the document with all sections, see nature.com/documents/nr-reporting-summary-flat.pdf

Life sciences study design

All studies must disclose on these points even when the disclosure is negative.

Sample size	Sample size was determined by the number of experimental repeats
Data exclusions	no data were excluded
Replication	Reproducibility was tested by repeating the experiments at least 3 times at different days/weeks.
Randomization	For some of the experiments the investigators were blinded (stated in the methods section). Most experiments did not need blinding as the readouts do not leave much space for biased interpretation.
Blinding	For the histological assessment of cell size and area HE sections, labels on the slides were coded in a way that did not allow the scientist to know the genotype.

Reporting for specific materials, systems and methods

We require information from authors about some types of materials, experimental systems and methods used in many studies. Here, indicate whether each material, system or method listed is relevant to your study. If you are not sure if a list item applies to your research, read the appropriate section before selecting a response.

Materials & experimental systems

n/a	Involved in the study	
☐	☒	Antibodies
☐	☒	Eukaryotic cell lines
☒	☐	Palaeontology and archaeology
☐	☒	Animals and other organisms
☒	☐	Clinical data
☒	☐	Dual use research of concern
☒	☐	Plants

Methods

n/a	Involved in the study	
☒	☐	ChIP-seq
☐	☒	Flow cytometry
☒	☐	MRI-based neuroimaging

Antibodies

Antibodies used	phospho-p38MAPK: Cell Signalling Technology, D3F9

p38MAPK: Cell Signaling Technology #9212
NFAT5 antibody: Thermo FIsher PA1-023
Agr2: Cell Signaling Technology AGR2 (D9V2F)

Validation

Agr2: https://www.cellsignal.com/products/primary-antibodies/agr2-d9v2f-xp-rabbit-mab/13062
phospho p38MAPK: https://www.cellsignal.com/products/primary-antibodies/phospho-p38-mapk-thr180-tyr182-d3f9-xp-rabbit-mab/4511
p38 MAPK: https://www.cellsignal.com/products/primary-antibodies/p38-mapk-antibody/9212
NFAT5: https://www.thermofisher.com/antibody/product/NFAT5-Antibody-Polyclonal/PA1-023

Eukaryotic cell lines

Policy information about cell lines and Sex and Gender in Research

Cell line source(s)

HepG2 cells: male
SW480 cells: male
Caco-2 cells: male
Capan-2 cells: male
MCF-7 cells: female
KOLF-2 cells: male

Authentication

HepG2: ATCC no. HB-8065, RRID:CVCL_0027
SW480: ATCC no. CCL-228, RRID:CVCL_0546
Capan-2: ATCC no. HTB-80, RRID:CVCL_0026
Caco-2: ATCC no. HTB-37, RRID:CVCL_0025
MCF-7: ATCC no. HTB-22, RRID:CVCL_0031
KOLF-2 (HPSI0114i-kolf_2-C1): hPSCreg: WTSIi018-B-1,Cellosaurus: CVCL_9S58

Mycoplasma contamination

all cell lines were tested monthly for mycoplasma contamination

Commonly misidentified lines
(See ICLAC register)

non of the cell lines used in this manuscript have been identified as misidentified cell lines. iclac.org/databases/cross-contaminations

Animals and other research organisms

Policy information about studies involving animals; ARRIVE guidelines recommended for reporting animal research, and Sex and Gender in Research

Laboratory animals

mus musculus, C57BL/6

Wild animals

n/a

Reporting on sex

mice were used gender matched, i.e. same numbers of females and ,ales

Field-collected samples

n/a

Ethics oversight

UK Home Office

Note that full information on the approval of the study protocol must also be provided in the manuscript.

Plants

Seed stocks

n/a

Novel plant genotypes

n/a

Authentication

n/a

Flow Cytometry

Plots

Confirm that:

- [ ] The axis labels state the marker and fluorochrome used (e.g. CD4-FITC).
- [x] The axis scales are clearly visible. Include numbers along axes only for bottom left plot of group (a 'group' is an analysis of identical markers).
- [ ] All plots are contour plots with outliers or pseudocolor plots.
- [ ] A numerical value for number of cells or percentage (with statistics) is provided.

Methodology

Sample preparation	no fluorochrome , native cells for FSC
Instrument	Attune NxT flow cytometer
Software	Attune Cytometric Software
Cell population abundance	cell lines
Gating strategy	no staining used, just cell lines

- [ ] Tick this box to confirm that a figure exemplifying the gating strategy is provided in the Supplementary Information.
